# Domestication and feedback: bidirectional hijacking in pancreatic ductal adenocarcinoma microenvironment

**DOI:** 10.3389/fimmu.2025.1585858

**Published:** 2025-08-11

**Authors:** Yuxi Qiao, Haobo Yin, Yong Zhang, Jingdong Zhang, Qian Dong

**Affiliations:** ^1^ Medical Oncology Department of Gastrointestinal Tumors, Liaoning Cancer Hospital and Institute, Cancer Hospital of China Medical University, Shenyang, China; ^2^ Pathology Department, Liaoning Cancer Hospital and Institute, Cancer Hospital of China Medical University, Cancer Hospital of Dalian University of Technology, Shenyang, China; ^3^ Medical Oncology Department of Gastrointestinal Tumors, Liaoning Cancer Hospital and Institute, Liaoning Key Laboratory of Gastrointestinal Cancer Translational Research, Cancer Hospital of China Medical University, Cancer Hospital of Dalian University of Technology, Shenyang, China

**Keywords:** pancreatic ductal adenocarcinoma, tumor microenvironment, cancer-associated fibroblasts, combination therapy, extracellular matrix

## Abstract

Pancreatic ductal adenocarcinoma (PDAC) is characterized by a tumor microenvironment (TME) composed of a dense extracellular matrix, cancer-associated fibroblasts (CAFs), vasculature, neural elements, and immune cell populations. This complex network promotes tumor proliferation, invasion, metastasis, and resistance to immunotherapy and chemotherapy. The microenvironmental characteristics of the various PDAC subtypes are discussed in this review. And we examines the role of cancer cells in the TME, highlighting their ability to manipulate stromal components to serve as collaborators in tumor progression. Furthermore, we explored the formation mechanism of the immunosuppressive microenvironment in PDAC, paying attention on Inflammation and intrinsic genetic alterations, the regulatory effect of metabolic reprogramming, the contribution of CAFs and the role of immune cells in cancer cell metastasis. This review shows the role of soluble molecules and exosomes in facilitating PDAC progression and immune evasion within the microenvironment. In conclusion, we outline the novel therapeutic strategies that focus on the interaction between cancer cells and their microenvironment, with the objective of offering new insights for future precision medical interventions.

## 1 Introduction

Pancreatic ductal adenocarcinoma (PDAC) is among the most lethal gastrointestinal malignancies, with an estimated five-year survival rate of 13% (1). According to the latest cancer statistics, it is the third leading cause of cancer-related deaths (1). By 2040, it is projected to rank as the second leading cause of cancer-related mortality globally (2). The mainstay of treatment for patients with resectable and borderline resectable PDAC with the goal of increasing R0 resection rates is surgery, supplemented by standard postoperative chemotherapy regimens. In addition, perioperative treatment of borderline resectable PDAC is now being explored (3); however, the asymptomatic onset and rapid disease progression of PDAC frequently result in late-stage diagnosis. Consequently, over 80% of patients present with locally advanced or metastatic stages, precluding them from potentially curative surgical intervention (4, 5). Even with standard surgical treatment, the one-year recurrence rate was up to 57.3% (6). Treatment for recurrent and metastatic PDAC includes chemotherapy, radiotherapy, and targeted therapy, with chemotherapy being the primary treatment option. Current first-line chemotherapy regimens such as FOLFIRINOX (oxaliplatin, irinotecan, folinic acid, and 5-fluorouracil), AG (albumin-bound paclitaxel plus gemcitabine), and gemcitabine monotherapy have a median survival of < 1 year in patients with metastatic PDAC who receive standard chemotherapy (7, 8). Immunotherapy has achieved good efficacy in many solid tumors and changed the current pattern of tumor treatment. However, achieving a breakthrough in PDAC treatment remains challenging, primarily owing to its unique tumor microenvironment (TME) (3, 9).

**Figure 1:**
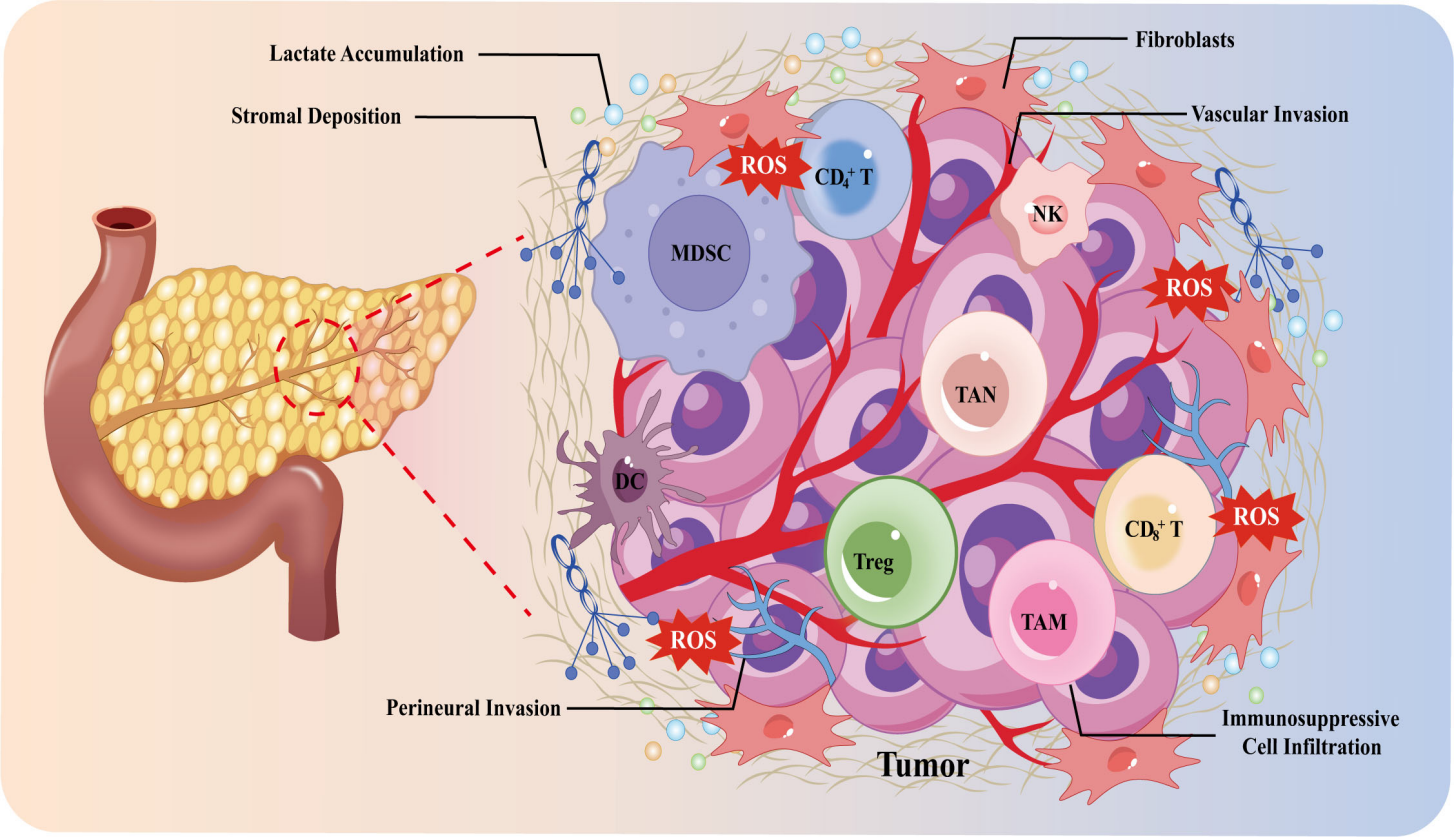
Overview diagram of the PDAC microenvironment and its hallmark features. DC cells, dendritic cells; MDSC, myeloid-derived suppressor cells; NK cells, natural killer cells; TAM, tumor-associated macrophage; TAN, tumor-associated neutrophil; Treg cells, regulatory T cells.

The TME of PDAC comprises a complex interplay of cancer-associated fibroblasts (CAFs), various immune cell subsets, extracellular matrix (ECM) components, vasculature, and neural elements (10), which collectively create a desmoplastic stroma that supports tumor progression and therapeutic resistance. The cellular composition and functional states within the TME are highly dynamic and can vary considerably depending on the genetic and phenotypic characteristics of the tumor cells, as well as the stage of disease progression (11). The hallmark features of the PDAC microenvironment include a dense fibrotic stroma, hypoperfusion, extensive perineural invasion (PNI), and profound immunosuppression, contributing to a “cold” immune milieu that impairs immune surveillance and antitumor responses. The immune-tolerant microenvironment of PDAC is a dynamic ecosystem primarily influenced by cancer cells that educate various stromal cells to actively contribute to tumor promotion. A comprehensive understanding of the PDAC microenvironment’s composition and the interaction mechanisms among multiple cellular components will significantly improve treatment strategies. The microenvironments of various PDAC subtypes differ, resulting in significant differences in therapy responses (12). Consequently, it is essential to establish a refined molecular subtyping of PDAC by integrating features from both the tumor epithelium and stromal microenvironment. This review focuses on the different subtypes of PDAC and their microenvironmental properties. We explores the intricate components of the PDAC–TME and highlights how PDAC cells actively reshape their surrounding microenvironment, exacerbating disease progression and establishing a distinct “cold” TME that differentiates PDAC from other solid tumors. Additionally, we examined the function of soluble molecules and exosomes in intercellular communication, along with novel therapeutic approaches aimed at the interaction mechanisms between cancer cells and their microenvironment (**Figure 1**).

## 2 PDAC subtypes

PDAC subtypes reveal the molecular characteristics of tumor cells and their significant association with the tumor microenvironment, encompassing immune cell infiltration, stromal components, and signaling pathways. Collisson et al. (12) initially categorized PDAC into three distinct subtypes: classical (CLA), quasimesenchymal, and exocrine-like. The CLA subtype exhibits high expression of adhesion-specific and epithelial genes, correlating with a favorable prognosis. The quasimesenchymal subtype exhibited elevated expression of mesenchymal genes and correlated with the poorest prognosis (12). The Moffit classification identifies two tumor-specific subtypes: the basal-like (BL) subtype and the CLA subtype. Furthermore, activated and normal matrix subtypes were identified based on specific matrix gene expression. Activated matrix subtypes are characterized by elevated expression of macrophage-related molecular genes, including Integrin subunit alpha M, C-C motif chemokine ligand 13(CCL13)and CCL18, as well as tumor-promoting secretory protein genes such as secreted protein acidic and rich in cysteine, gelatinase B (MMP9), and stromal hemolysin 3 (MMP11) (13). Studies indicate that the epigenetic regulatory factor bromodomain-containing protein 4 enhances the expression of the transcription factor cellular Jun proto-oncogene, which in turn promotes tumor cells to secrete CCL2, facilitating macrophage recruitment. The inflammatory factor tumor necrosis factor-alpha(TNF-α), secreted by macrophages, can activate cJUN/Activator protein 1(AP1), leading tumor cells to transition from the CLA subtype to the BL subtype, which is associated with a poorer prognosis (14). The intervention with TNF-α resulted in a significant reduction of CD3^+^, CD4^+^, and CD8^+^ T cell infiltration in the tumor microenvironment, alongside alterations in the tumor compartments (15). Puleo et al. (16) improved the original Moffit classification by introducing five subtypes: pure basal like, stroma activated, desmoplastic, pure classical, and immune classical. The expression of the Focal Adhesion Kinase(FAK) pathway was significantly enriched in both the desmoplastic type and the stroma activated type (16). The effective treatment of PDAC necessitates the departure from conventional single pathological classification models. It is essential to develop a multi-classification system that incorporates the characteristics of tumor epithelium and microenvironment heterogeneity. These PDAC subtype classifications enhance the understanding of microenvironment characteristics and treatment response mechanisms across different subtypes. **Table 1** summarizes the different classifications of PDAC subtypes and their features.

**Table 1:** Different classifications of PDAC subtypes and their features.

Classification name	Subtypes	Features
Collisson 2011	classical	High expression of epithelial genes, and associated with favorable prognosis
	quasi-mesenchymal	High expression of mesenchymal genes, and associated with the worst prognosis
	exocrine-like	Associated with the expression of digestive enzyme-related genes
Moffitt 2015	classical	The best prognosis, unable to benefit from postoperative adjuvant therapy.
	basal	Poor prognosis, benefit from postoperative adjuvant therapy
	normal	Upregulation of markers such as pancreatic stellate cell and smooth muscle actin
	activated	Activated inflammatory stromal response, poor prognosis
Bailey 2016	pancreatic progenitor	Associated with the classical subtype
	acinar-derived exocrine	Associated with the exocrine subtype
	squamous	Inflammatory and hypoxic gene expressions are in an activated state.
	immunogenic	Upregulation of CTLA-4 and PD-1
Puleo 2018	pure basal-like	Predominantly characterized by tumor cell features.
	stroma-activated	Sensitive to FAK inhibitors;Low immune cell infiltration with high content of fibroblasts and endothelial cells.
	desmoplastic	The highest infiltration of all immune cells, with significant activated stromal signals.
	pure classical	Low overall cell infiltration rate
	immune classical	Predominantly infiltrated by natural killer (NK) cells, T cells, and B cells, with low levels of activated stroma.

## 3 CAFs and tumor cells: heterogeneity and plasticity

Fibroblasts are crucial in tissue homeostasis, wound healing, inflammation, fibrosis, and ECM synthesis (17, 18). During carcinogenesis, repeated accumulation of Kirsten Rat Sarcoma (KRAS) mutations and Small mother against decapentaplegic 4 (Smad4*)* deletion can induce transforming growth factor beta (TGF-β) signaling activation and secretion of interleukin (IL)-33 in the stroma, contributing to the transformation of fibroblasts or pancreatic intraepithelial neoplasia-associated fibroblasts into CAFs (19–22). A pivotal event in PDAC initiation is acinar-to-ductal metaplasia (ADM). This process manifests as a transient, reparative plasticity during pancreatic inflammation or injury, yet undergoes irreversible neoplastic progression when occurring in acinar cells with accumulated *KRAS* mutations, ultimately evolving into PDAC (23, 24). Seema et al. (23) identified a novel laminin α5/integrin α4/activator of transcription 3(STAT3) axis mediated by CAFs, demonstrating its critical role in facilitating ADM during PDAC progression. The studies indicate that in the early stages of PDAC, a reciprocal reprogramming mechanism exists between tumor cells and CAFs. Tumor cells promote CAF differentiation through oncogenic signaling (TGF-β/IL-33), while CAFs encourage the malignant transformation of acinar cells, establishing a self-reinforcing positive feedback loop. CAF activation involves multiple downstream signaling pathways such as the sonic hedgehog (SHH) pathway, Janus kinase/signal transducer(JAK)/STAT3 and nuclear factor kappa-light-chain-enhancer of activated B cells(NF-κB)signaling pathways (25). The STAT3 transcription factor promotes the intrinsic activation of CAFs and serves as a key mediator in the inflammatory regulation during ADM (25, 26). Consequently, targeting these pathways (e.g., via STAT3 inhibitors) represents a promising therapeutic strategy to concurrently suppress CAF activity and ADM progression. CAFs are the most abundant cell type in PDAC and a key promoter of the desmoplastic reaction through excessive ECM deposition, thereby increasing tissue rigidity. Concurrently, they enhance interstitial fluid pressure and restrict angiogenesis, limiting tumor growth while contributing to chemoresistance (27–29). In nutrient-deprived conditions, CAFs secrete various metabolites that support tumor cell proliferation (30, 31). For instance, elevated expression of the glutamatergic presynaptic protein Netrin G1 in CAFs upregulates glutamate, glutamine, and cytokine expression, sustaining tumor cell viability through direct cell-cell interactions or activation of the Netrin G1/Netrin-G-Ligand-1 signaling pathway (30, 31). CAFs can secrete not only amino acids such as glutamine and glutamate, but also lactic acid and fat intermediates. Furthermore, recent findings by Divya et al. (32) revealed that CAF can secrete acetate, which on one hand provides carbon raw material for energy metabolism of cancer cells. On the other hand, CAF can remodel histone acetylation including Histone H3 lysine 9 (H3K9),H3K18,H3K27 through acetyl-coa synthase family enzymes to support the survival of cancer cells in acidic conditions.

The high heterogeneity in phenotype and function among CAF subsets, coupled with their diverse cellular origins, profoundly influences tumor trajectory and therapeutic response. Resident quiescent pancreatic stellate cells (PSCs) are conventionally viewed as the primary source of CAFs in PDAC (33). Lineage tracing and ablation studies reveal that PSCs contribute minimally to CAFs in PDAC, with the transformation frequency influenced by tumor genotype (34). Monocytes, macrophages derived from bone marrow, mesenchymal stem cells derived from adipose tissue, endothelial cells, mesothelial cells, and pericytes are among the CAF precursors in PDAC, according to additional lineage-tracing investigations into CAF origins (19, 35–39). Notably, CAFs transformed under different stressors or intratumoral factors may exhibit functional preferences that impact their roles. Recent research demonstrates that tumor-intrinsic deficiency of SET domain containing 2, a histone lysine methyltransferase, drives transcriptional reprogramming through aberrant H3K27 acetylation deposition and promotes bone morphogenetic protein 2(BMP2) signaling pathway activation. This epigenetic alteration promotes the differentiation of pancreatic stellate cells (PSCs) and bone marrow-derived mesenchymal stem cells into a lipid-rich phenotype. These lipid-enriched CAFs, characterized by co-expression of ATP-binding cassette subfamily A member 8 (ABCA8) and fibroblast activation protein (FAP) (ABCA8^+^FAP^+^), promote tumor metabolism through ABCA8-mediated lipid transfer to fuel mitochondrial oxidative phosphorylation (OXPHOS) (40). The isolation of CAFs presents a considerable challenge in contemporary research, primarily due to the lack of specific biomarkers. Research indicates that CAFs in PDAC co-express various structural proteins, including α-smooth muscle actin (α-SMA), fibroblast-specific protein 1 (FSP1), also referred to as S100 calcium-binding protein A4 (S100A4), (FAP), and platelet-derived growth factor receptor α/β (PDGFRα/β). Nonetheless, the majority of markers are common across various cell types. A variety of transcription factors, such as paired related homeobox 1 (Prrx1), STAT3, and Yes-associated protein 1 (YAP1), play a crucial role in determining CAF phenotypes. Additionally, their significant plasticity complicates the definitive identification of CAFs (19). Three classical subtypes of CAFs have been defined: (1) myofibroblastic CAFs (myCAFs), marked by high α-SMAexpression; (2) inflammatory CAFs (iCAFs), characterized by IL-6 and IL-10 secretion; and (3) antigen-presenting CAFs (apCAFs), expressing major histocompatibility complex class II and CD74 (36, 41). Functionally, iCAFs are localized within the dense stroma distal to tumor cells and exhibit immunosuppressive and pro-tumorigenic properties. In contrast, myCAFs are typically involved in suppressing tumor growth and immune responses. However, recent studies indicate that myCAFs exert context-dependent effects on PDAC cells. Gianluca et al. (42) demonstrated that PDAC cells secrete TGF-β, which activates the epidermal growth factor receptor pathway in myCAFs, significantly enhancing the metastatic potential of malignant PDAC cells. Consistent with these findings, Ge et al. (43) reported that epidermal growth factor receptor-driven myCAF reprogramming facilitates epithelial-mesenchymal transition (EMT), correlating with poor prognosis. Additionally, CAFs promote early PDAC dissemination through the SRY-Box transcription factor 4-matrix metalloproteinase 1 (MMP1) signaling cascade. Ela et al. (44) discovered that IL-1 and TGF-β can induce mesothelial-to-apCAFs transdifferentiation, thereby enabling apCAFs to promote the differentiation and expansion of cluster of differentiation CD4^+^ T cells into regulatory T cells (Tregs) through antigen-specific mechanisms, contributing to immune evasion. Single-cell technologies have facilitated the discovery of various novel CAF subtypes. Chen et al. (45) utilizing single-cell RNA sequencing (scRNA-seq) and Weighted Gene Co-expression Network Analysis, discovered a complement-secreting CAF subpopulation (csCAFs).This subtype predominantly occupies periductal stromal regions near malignant ducts in early-stage PDAC, exhibiting marked complement system activation with elevated expression of complement factors including complement component 3 (C3), C7, complement factor B (CFB), CFD, CFH, and CFI, enabling modulation of immune and inflammatory responses (45). Wang et al. (46) identified metabolically activated CAFs (meCAFs) in low-connectivity PDAC tumors, characterized by a hyperactive metabolic state with overexpression of phospholipase A2 group IIA (PLA2G2A) and cellular retinoic acid binding protein 2 (CRABP2). Patients harboring this subtype demonstrate increased metastatic risk but significantly improved response to immunotherapy. Mizutani et al. (47) reported tumor-suppressive Meflin-positive CAFs that improve PDAC outcomes. Lineage tracing confirms Meflin^+^ cells generate α-SMA^+^ CAFs (47). Recently, Sun et al. (48) employed scRNA-seq and multiplex immunohistochemistry to identify fibroblast activation FAPα^+^CD144^+^ endothelial-like CAFs (endoCAFs). These FAPα^+^CD144^+^ endoCAFs acquire vasculogenic mimicry(VM)capabilities to facilitate metastasis while promoting *in situ* tumor proliferation and invasion via the CD144-β-catenin-signal transducer and STAT3 signaling axis, exerting dual pro-tumorigenic functions (48). Other studies revealed leucine-rich repeat-containing protein 15-positive (LRRC15^+^) myofibroblasts whose development depends on TGF-βreceptor 2 signaling. These CAFs correlate with poor response to PD-L1 immune checkpoint blockade (ICB) (49). **Table 2** summarizes representative biomarkers and functions of heterogeneous CAF subtypes in PDAC.

**Table 2:** Summarizes representative biomarkers and functions of heterogeneous CAF subtypes in PDAC.

Subtypes	Markers	Functions
myCAF	αSMA, FAP, FSP1, PDGFR-α, Col 1, MYL, TGF-β1, ACTA2,PDPN	Produce ECM, activate immune responses, mediate EMT, and release factors that modulate angiogenesis and metastasis.
iCAF	FAP,IL-6,IL-11, CXCL1/2/12	Mediate immunosuppression and promote tumor growth
apCAF	MHCII,CD74, FSP1, SAA3	Induce differentiation and expansion of CD4^+^ T cells into Tregs, antigen presentation
meCAF	PLA2G2A,CRABP2	Participate in glycolysis to provide OXPHOS in cancer cells, improving immunotherapy but worsening prognosis.
LRRC15^+^CAF	αSMA,TGF-β,LRRC15	Poor response to immunotherapy
Meflin ^+^ CAFs	Meflin, αSMA^low^	Generate α-SMA^+^ CAFs and exert tumor-suppressive functions
csCAFs	C3,C7,CFB,CFD,CFH,CFI	May modulate intratumoral immune and inflammatory responses
endoCAFs	FAPα 、CD144	VM forms vascular-like channels which promote tumor cell metastasis and paracrine signaling encourages local tumor cell proliferation and invasiveness.
ABCA8^+^FAP^+^ CAFs	ABCA8,FAP, PDPN, CXCL14, Lp1	Link to tumor cell lipid metabolism.
NetG1^+^CAF	αSMA, PDPN, NetG1	Supply amino acids to sustain cancer cells, while evading the cytotoxic effects of NK cells.

These newly identified CAF subpopulations reflect the complex plasticity and dynamic nature of CAFs within the TME. For example, iCAFs and myCAFs can transdifferentiate under specific cytokine signals; IL-1 drives iCAF formation, whereas TGF-β suppresses IL-1 receptor expression, inducing conversion to myCAFs (50). In PDAC, CAFs highly express tyrosine kinase inhibitor 1 (TKI1)molecules, and the presence of TKI1^+^ also contributes to the conversion of myCAFs to iCAFs (51). Hypoxic conditions within the dense fibrotic stroma may also promote iCAF polarization (52). Feldmann (53) identified the Prrx1 as a key regulator of EMT and metastasis, mediating the phenotypic switching of CAFs between quiescent and activated states, further underscoring the phenotypic plasticity of CAFs in PDAC.

The formation and polarization of CAF may be involved in the occurrence and development of PDAC. Targeting CAF to participate as a crucial molecule in the origin and progression of PDAC may reshape the microenvironment of PDAC. However, its high heterogeneity and plasticity result in some challenges in the precise targeting of CAF. One of the current treatments is to inhibit the CAF activation pathway, including SHH, JAK/STAT3, and TGF-β inhibitors. However, SHH pathway inhibition changed the proportion of myCAF/iCAF fibroblasts in PDAC and increased the proportion of immunosuppressive iCAF instead (54). FAK inhibitors can reduce tumor metastasis and reshape ECM (55). Targeting C-X-C motif chemokine ligand 12 (CXCL12) and CXC receptor (CXCR4) inhibits iCAF activation and enhances the immune response in PDAC (56). Additionally, different treatment methods, such as targeted gene mutation and reprogramming CAF, are gradually carried out in clinical trials. The successful benefits of the trial also require clear and appropriate drug compatibility and accurate screening of the target population.

## 4 ECM and cancer cells: enemies or friends?

Connective tissue hyperplasia is a characteristic tissue marker of PDAC. The ECM constitutes the predominant stroma in PDAC, characterized by an intricate network composed of collagen, proteoglycans, proteases, growth factors, and chemokines (57). The relative proportions of ECM components and tumor cells within PDAC can significantly influence tumor biology. For example, tumors with excessively reduced collagen content exhibit shorter overall survival rates (58). Beyond the tumor matrix composition, the mechanical properties of the ECM also impact EMT, metabolic changes, invasion, and tumor cell metastasis (59). A rigid ECM can dampen cGAS immune signaling by activating the Rho-associated protein kinase-myosin II-F-actin signaling pathway in tumor cells, subsequently modulating tumor immunogenicity (60). In addition, cancer cells can sense mechanical stress in the matrix and enhance the Warburg effect to promote glycolysis-dependent tumor growth (59). In contrast, softened ECM can mediate Yes-Associated Protein 1 degradation through the autophagic lysosomal pathway, leading to cancer cell dormancy (61). Notably, the ECM is traditionally viewed as a tumor-promoting entity; however, it may also possess protective properties that inhibit tumor progression (27). Evidence indicates that targeting lysyl oxidase-like-2 with specific antibodies reduces ECM content, accelerates tumor progression, and correlates with decreased overall survival (62). Chen et al. (63) further elucidated the protective role of the ECM, noting that fibrocollagen–the most abundant matrix component in PDAC–comprises approximately 80% of the total ECM. Their study demonstrated that a decrease in type I collagen content within fibrocollagen upregulated CXCL5 in cancer cells, leading to the recruitment of myeloid-derived suppressor cells and inhibition of CD8^+^ T cell activity, ultimately exacerbating PDAC progression and diminishing overall survival (63). In the stroma, binding of the non-fibrillar collagen type XV to discoidin domain receptor1 and e-cadherin also reduces PDAC invasion and metastasis (64).

Proteomic analyses reveal that, although stromal cells produce approximately 90% of the ECM, a portion is synthesized by cancer cells themselves (57). Notably, the protective effects of type I collagen secreted by cancer cells mirror those produced by CAFs. During PDAC progression, fibrous collagen becomes progressively enriched and maintains its procollagen C domain.BMP1, which specifically cleaves procollagen I derived from cancer cells, facilitates type I collagen deposition and inhibits tumor growth (65). Conversely, other stromal components secreted by cancer cells have been implicated in promoting metastatic behavior. For instance, three stromal proteins–agrin, serine protease inhibitor B5, and cystatin B–are involved in various stages of metastasis, including EMT, pseudopodia formation, and extravasation of PDAC cells (66). Remarkably, cancer cells may exploit the mechanical properties of the ECM, enhancing its stiffness and contributing to a positive feedback loop. Recent investigations by Pierluigi et al. (67) identified transitional morphobiotype cancer cells associated with collagen network reorganization, potentially leading to collagen deposition and increased ECM rigidity. These findings suggest that cancer cells may modulate the surrounding ECM through direct and indirect mechanisms.

These findings suggest that tumor cells may regulate the ECM through direct and indirect mechanisms, and ablation of stromal deposits alone does not prolong overall patient survival, considering the dual role of the ECM in cancer progression (68). The current therapeutic dilemma faced by targeted ECM is mainly due to its dense nature and the interconnections between specific components of the microenvironment, which ultimately act as a drug barrier and immunosuppression. Softening the matrix to alter its mechanical properties, as well as targeting specific matrix components to remodel the cancer-suppressive ECM and the use of nanomaterials in bioengineering will help increase tumor drug delivery.

## 5 Vascular endothelial cells and cancer cells: nutrition support and metastasis

In 1971, it was first proposed that tumor cells rely on blood supply to obtain oxygen and nutrients needed for growth, a process termed tumor angiogenesis (69). This neovascularization is often structurally and functionally aberrant, resulting in interstitial hypertension, hypoxia, and acidosis, which create a TME that facilitates tumor proliferation, invasion, and metastasis (70). The hypoxia-inducible factor is a key mediator of cellular response to hypoxia and activates the transcription of pro-angiogenic factors, including vascular endothelial growth factor (VEGF), PDGFB, MMP-2, and MMP-9 (71). Tumor cells secrete VEGF to promote the migration and proliferation of vascular endothelial cells, enhancing microvascular permeability and ultimately driving tumor angiogenesis However, recent research suggests that angiogenesis in PDAC may be independent of VEGF signaling. For instance, PDAC can promote tumor progression through non-VEGF-dependent angiogenesis, mediated by the Bicaudal C Homolog 1/Lipocalin 2 axis, highlighting novel therapeutic targets for anti-angiogenic strategies.

Additionally, PDAC cell-derived exosomal micro ribonucleic acids (RNAs) have emerged as key modulators of angiogenesis. For example, exosomal miR-30b-5p promotes angiogenesis by downregulating gap junction protein 1 under hypoxic conditions (72). Similarly, cancer cell-derived exosomal miR-27a has been shown to regulate angiogenesis by influencing human microvascular endothelial cell function (73, 74). Beyond classical angiogenesis, tumor cells can facilitate neovascularization through non-angiogenic pathways, such as VM (75) and vascular co-option (76). VM is associated with various signaling pathways, including Notch signaling (77) and the extracellular signal-regulated kinase 1/2/2-MMP-2/9 axis (78). Under hypoxic conditions, hypoxia-inducible factor-2α(HIF-2α) can further promote this phenomenon (79). These findings indicate that tumor cells can induce angiogenesis through various mechanisms to achieve feeding. The non-response of PDAC to anti-angiogenic therapy may be related to non-angiogenic pathways in PDAC cells.

Cancer-associated neovascularization provides essential nutrients and oxygen to support tumor growth and establishes potential routes for metastasis. Before metastasis dissemination occurs, a decrease in the number of pericytes surrounding microvessels in pre-metastatic niches leads to the loss of integrity between endothelial cells and the basement membrane. This results in the formation of highly permeable, immature blood vessels that facilitate the intravasation and dissemination of cancer cells to distant metastatic sites (80).

In summary, tumor cells form cancer neovasculature to provide access to nutrients and metastasis through a variety of mechanisms. However, antivascular therapy targeting VEGF has not shown benefit in previous clinical trials in PDAC (81). This may be due to the fact that angiogenesis in PDAC involves multiple bypass activation, which requires anti-angiogenic drugs in combination with other drugs to cover each signaling pathway. In contrast, the process of vascular provision of nutrients in PDAC may differ from other solid tumors and not depend on neoangiogenesis, which needs to be further understood in conjunction with the mechanisms of nutrient metabolism in tumor cells. In addition, some therapeutic directions, such as targeting non-angiogenic pathways may bring new therapeutic opportunities for PDAC.

## 6 Nerves and cancer cells: PNI

A genetically engineered mouse model of PDAC has shown that the nervous system plays a role in all stages of cancer development, including the precancerous stage (82). PNI is a hallmark feature of PDAC and is present in 70–100% of cases (83, 84). PNI is associated with pain, increased tumor aggressiveness, and a higher propensity for locoregional spread, thereby serving as a key prognostic factor for tumor recurrence and overall survival (85). PNI indicates a unique interaction where cancer cells exploit neural structures to facilitate tumor progression. Cancer cells promote nerve growth and guide cancer cell migration along neural tracts by secreting nerve growth factors (NGF), neurotrophic factors, and chemokines (86, 87). A therapeutic target that inhibits this process has recently emerged. NGF activates the pro-myosin receptor kinase (Trk), and Lar@NP-OMVs (which contain Trk inhibitors) directly disrupt neural activity by inhibiting the neurotrophic factor/Trk signaling pathway and converting M2-type tumor-associated macrophages (TAMs) to M1-type and enhancing the efficacy of gemcitabine (88). Vera et al. (89) employed novel tracing technology Trace-n-Seq and single-cell transcriptomics to reveal how PDAC cells co-opts the nervous system. Their study revealed that in PDAC, cancer cells reprogram neurons, resulting in significant neurite outgrowth and their conversion into neurofilament subtype sensory neurons. Moreover, the study established a distinct pancreatic cancer neural gene signature by integrating robustly replicated differentially expressed genes across five key sympathetic and sensory neuronal subpopulations. This signature persists after tumor resection and may be associated with tumor proliferation and local recurrence (89). Moreover, co-opted nerves become pro-tumorigenic allies in PDAC. Beyond participating in early tumorigenesis (82), sensory nerves secrete CCL21 and CXCL10, which chemoattract PDAC cells toward sensory neurons and exacerbate cancer-associated pain (90). Beyond sensory nerves, the pancreas receives dual innervation from peripheral motor nerves—specifically the sympathetic and parasympathetic nervous systems. However, their roles in pancreatic cancer progression exhibit antagonistic effects. Sympathetic nerves can release catecholamines that suppress CD8^+^ T cell activity and promote tumor progression (91). Studies in mouse models revealed that subdiaphragmatic vagotomy accelerates tumor progression. Further investigation demonstrated that cholinergic signaling suppresses tumorigenesis through Mitogen-Activated Protein Kinas(MAPK)pathway and Phosphoinositide 3-Kinase/Protein Kinase B (PI3K/AKT)pathways (92). However, another study revealed that acetylcholine affects cancer cells in a dose-dependent manner. Excessive acetylcholine suppresses interferon-gamma (IFNγ) production by CD8^+^ T cells and promotes T cell differentiation toward the Th2 phenotype (93).Furthermore, in terms of metabolic reprogramming, nerve cells may provide an alternative nutrient source to sustain tumor growth. For instance, Robert et al. (94) found that in a nutrient-deficient PDAC microenvironment, neuronal axons supply serine, promoting tumor cell proliferation. Remarkably, this process reflects the neurotrophic recruitment by cancer cells: serine deprivation induces ribosomal stalling specifically at two of the six serine codons (TCC and TCT), thereby driving PDAC cells to selectively translate and secrete NGF, promoting tumor innervation (94). In turn, neurons secrete glutamate, which binds to ionotropic glutamate receptors, leading to calcium influx and activation of the downstream Ca^2+^/calmodulin-dependent protein kinase II–ERK/MAPK signaling pathway. Subsequently, this cascade upregulates hexokinase 2 expression via N^6^-methyladenosine modification, ultimately enhancing tumor glycolysis (95). In PDAC, intraneural invasion occurs when cancer cells infiltrate the endoneurium–the innermost nerve layer, composed predominantly of axons and Schwann cells (SCs) (96). SCs, critical for PNI, can promote PDAC cell proliferation by transforming into a non-myelinating phenotype through c-Jun-mediated reprogramming (97). This reprogramming pathway is termed the tumor-activated SC trajectory. SCs exert mechanical forces that facilitate cancer cell invasion along these neural paths (97). Recently, Tian (98) discovered a paracrine feedback loop between SCs and tumor cells. Tumor-derived tissue inhibitor of metalloproteinase 1 (TIMP1) promotes SC proliferation and migration through the CD63/PI3K/AKT pathway. In turn, SCs secrete CCL7, which enhances cancer cell migration, invasion, and TIMP1 expression through C-C chemokine receptor(CCR2)/STAT3 signaling. Silencing TIMP1 *in vitro* and *in vivo* disrupted this paracrine signaling (98), suggesting a potential therapeutic target for inhibiting PNI in PDAC. Autophagy is a primary degradation and recycling mechanism that delivers various cellular materials to lysosomes. It and its effector mechanisms are increasingly recognized as critical players in cancer development and advancement (99). Cancer cell-derived NGF induces SC autophagy, which promotes a nerve repair-like response, thereby enhancing autophagic activity in tumor cells. Combined inhibition of NGF and autophagy (e.g., chloroquine/hydroxychloroquine) suppresses PNI initiation and progression in pancreatic cancer (100).

In PDAC, CAFs serve as the most prominent interaction partners of neurons. *In vitro* co-culture and neuron-conditioned medium experiments demonstrate that neurons directly enhance CAF proliferation (by 30–50%) via secreted soluble factors IL6. RNA-seq reveals that neurons activate MYC target genes and G2/M checkpoint pathways in CAFs, promoting a tumorigenic phenotype (89). Recent research has revealed that PDPN^+^PDGFRα^+^ CAFs release specific long non-coding RNAs (lncRNAs) via extracellular vesicles. These lncRNAs mediate 5-methylcytosine modification of tumor cell RNA, thereby stabilizing mRNA expression of PNI-related genes and significantly enhancing cancer cell neurotropism (101). Furthermore, this specific lncRNA promotes TNF-α secretion by tumor cells, which activates PDPN^+^PDGFRα^+^ CAFs through the NF-κB pathway, forming a feedforward loop that amplifies neural invasion (101). In Li et al.’s study, researchers isolated PNI-associated CAFs and uncovered their critical role in tumor metabolic reprogramming. These CAFs secrete lactate that induces histone H3K18 lactylation, thereby activating transcription of neural invasion-related genes, ultimately driving PNI in PDAC (102).

Collectively, these studies suggest that PNI is a dynamic, bidirectional interaction between nerves and cancer cells, forming a specialized microenvironment that facilitates aggressive tumor behavior and metastasis. High neural infiltration in PDAC correlates with increased pain and heightened metastatic potential, underscoring the need to further elucidate the mechanisms of neural regulation in cancer. A clear understanding of the mechanisms of cancer cell-neuronal cell interactions and the causal associations of various pathways will bring breakthroughs needed to achieve precision-targeted PDAC therapy.

## 7 Multidimensional regulation of the tumor immunosuppressive microenvironment

### 7.1 Intrinsic genetic mutations and inflammation

Genetic mutations are critical drivers of PDAC tumorigenesis and significantly influence the immune landscape. The most commonly mutated gene in PDAC, include *KRAS*), *Tumor Protein 53(TP53)*, *SMAD4*, and *Cyclin-Dependent Kinase Inhibitor 2A* (103). Recent evidence indicates that oncogenic mutations can remodel the TME. The majority of pancreatic intraepithelial neoplasia(PanIN)lesions carry oncogenic *KRAS* mutations that drive pancreatic tumorigenesis (104). In early-stage PanIN, Kras^G12D^mediates upregulation of granulocyte-macrophage colony-stimulating factor(GM-CSF), stimulating expansion of Gr1^+^CD11b^+^myeloid-derived suppressor cells(MDSCs)while reducing CD8^+^ T cell infiltration (105). Liu et al. (104) identified a pivotal accelerator for *KRAS*-mutant PanIN progression to PDAC: peroxisome proliferator-activated receptor-δ (PPARδ), which is upregulated in both human and murine PanIN. Under high-fat diet stimulation, PPARδ activation prompts *KRAS^G12D^* ^-^bearing PanIN to secrete CCL2. Via the CCL2/CCR2 axis, this chemokine recruits immunosuppressive macrophages and MDSCs into the pancreas, thereby accelerated PDAC development (104). Moreover, recruited M2-polarized macrophages can release the inflammatory cytokine IL-1β, facilitating early inflammatory reprogramming in PDAC and so accelerating the onset of pancreatic cancer (106). Chronic pancreatitis is a known risk factor for PDAC development, and repeated inflammatory insults in murine models accelerate tumor initiation and metastatic spread. Inactivation of the STAT3, a central inflammatory mediator, can prevent the progression of PanIN to PDAC (26, 107, 108). A positive feedback loop between tumor cells and IL-1β-expressing TAMs further exacerbates the persistence of inflammation. Tumor cell-derived prostaglandin E2 (PGE2) and tumor necrosis factor induce TAMs to secrete IL-1β, which in turn enhances PGE2 production and tumor necrosis factor in cancer cells, perpetuating a pro-inflammatory state. Disruption of the PGE2–IL-1β axis has been shown to reprogram TAMs towards an anti-tumorigenic phenotype, thereby attenuating tumor growth (106). PDAC cell-derived debris can activate M2-polarized TAMs to secrete IL-1β through the Toll-like receptor 4/IL-1R domain-containing adaptor-inducing IFN-β and NF-κB signaling pathways (109). He et al. (110) showed that gene mutations affect the conversion of immune-activated cells into immunosuppressive cells. Similarly, Kras^G12D^ mutations upregulate IL-10 and TGF-β, promoting the conversion of CD4^+^CD25^+^T cells into immunosuppressive Tregs (110). Not only the Kras^G12D^ mutation, but also the Kras^G12V^mutation has been found to correlate with elevated levels of Tregs in the circulation (111). The *KRAS ^G12D^* mutation, the predominant variant within the *KRAS* gene family (103), constitutes a viable therapeutic target deserving investigation. Furthermore, inhibiting KRAS^G12D^ mutant protein with MRTX1133 has been shown to reverse early PDAC lesions, reduce tumor burden, and promote CD8^+^ T cell-mediated cytotoxicity by inducing FAS expression, which increases CD8^+^ T cells in tumors and reprograms CAFs (112). These findings highlight the complex role of oncogenic mutations in shaping the PDAC immune microenvironment. Mutations in the tumor suppressor gene *TP53* are observed in 80% of PDAC, with the majority being missense mutations associated with allelic loss (103, 113). TP53 mutation induces loss of the tumor suppressor *ETS* homologous factor (EHF, epithelium-specific *ETS* transcription factor) and activates the CXCL1-CXCR2 axis, thereby promoting recruitment of immunosuppressive CXCR2^+^ neutrophils. In preclinical studies, the combination of nitrofurantoin (a pharmacological agent restoring EHF expression) with anti-PD-1 antibody and gemcitabine (GEM) markedly suppressed tumor growth, demonstrating significant translational therapeutic potential (113). Furthermore, compared to tumors harboring KRAS^G12D^ mutation alone, the co-mutation of TP53 and KRAS^G12D^ induces an immunosuppressive tumor microenvironment characterized by a reduced T helper 1(Th1)/Th2 cell ratio, elevated Treg infiltration, and an increased Treg-to-tumor-specific CD4^+^ T cell ratio, collectively contributing to significantly poorer survival rates (114).

### 7.2 Epigenetics and Metabolic Reprogramming

Cancer cells predominantly rely on glycolysis for ATP generation, even under aerobic conditions—a metabolic reprogramming termed the Warburg effect (115). This adaptive mechanism of cancer cells leads to higher rates of glucose metabolism and lactate production, resulting in lactate accumulation and providing an acidic environment for cancer cell proliferation and immune escape (116). Hexokinase 1/2 (HK1/2) and lactate dehydrogenase A (LDHA), both associated with lactate synthesis, are overexpressed in neoplastic cells. Lactate generated during metabolism can serve as a substrate for histone modification, facilitating histone lactoacylation to modulate LDHA (115, 117). Nucleolar and spindle-associated protein 1 (NUSAP1), a microtubule-binding oncoprotein, forms a transcriptional complex with cellular Myelocytomatosis viral oncogene homolog (c-Myc) and HIF-1α on the LDHA promoter, amplifying its expression. Notably, lactate stabilizes NUSAP1 via lysine lactylation, creating a self-reinforcing loop that drives glycolytic flux and further elevates NUSAP1 levels (118). H3K4 and H3K18 undergo lactoylation (H3K4la/H3K18la), enhancing transcription of TTK protein kinase (TTK) and BUB1 mitotic checkpoint serine/threonine kinase B (BUB1B). TTK activates LDHA, increasing lactate production and further promoting histone lactoylation, thereby establishing a glycolysis-H3K18la-TTK/BUB1B feedforward loop. This self-reinforcing mechanism amplifies the tumor’s lactic acid-rich microenvironment (117). Cancer cells critically depend on amino acid metabolism for nutrient acquisition. Among these, glutamine—the most abundant non-essential amino acid in circulation—plays a central role. Its carbon backbone fuels the tricarboxylic acid (TCA) cycle as an anaplerotic substrate, while its nitrogen moiety supports biosynthesis of amino acids, hexosamines, and nucleotides, sustaining proliferation and metabolic reprogramming (119). Cancer cells can remodel glutamine metabolism to maintain reduction-oxidation reaction homeostasis through non-classical pathways (120). Experimental evidence indicates that acute glutamine restriction suppresses tumor cell proliferation, whereas chronic depletion induces adaptive metabolic reprogramming to sustain survival (121). To sustain proliferation under glutamine deprivation, cancer cells upregulate glutamine ammonia ligase (GLUL, also known as glutamine synthetase) through c-Myc-driven transcriptional activation and epigenetic modulation. Furthermore, GLUL can alternatively utilize α-ketoglutarate and ammonium as precursors for glutamine synthesis, maintaining metabolic flexibility (122). Moreover, glutamine deprivation enhances trimethylation of H3K4, upregulates key ferroptosis inhibitors including solute carrier family 7 member 11 and glutathione peroxidase 4, thereby suppressing lipid peroxidation(LPO) and ultimately conferring ferroptosis resistance in cancer cells (121). Beyond glucose and glutamine metabolism, cancer cells also exhibit dysregulated lipid metabolism to sustain membrane biosynthesis, energy storage, and signaling for diverse cellular activities (123). Cancer cells upregulate key lipogenic enzymes—acetyl-CoA carboxylase, ATP-citrate lyase, and sterol O-acyltransferase 1—to promote cholesterol and lipid biosynthesis. Leveraging fatty acids as metabolic substrates, they sustain redox balance and fuel proliferation and metastatic progression (123). Previous studies have found that in PDAC, the intermediate products of glutamine involved in the tricarboxylic acid cycle can be used by fatty acid synthase (FASN) to generate fatty acids, and c-Myc, in cooperation with KRAS and HIF1A, can induce the expression of related enzymes (124). Additional studies suggest that the histone lysine (K)-specific methyltransferase 2, a modulator of metabolic gene expression, contributes to the transcriptional regulation of FASN (125). In addition, c-Myc can upregulate ELOVL fatty acid elongase 6, a c-MYC-regulated fatty acid elongase, to drive lipid synthesis (124). Notably, cancer cells fuel their rapid proliferation by depriving the microenvironment of nutrients such as glucose, amino acids, and lipids. This creates a metabolic niche characterized by high lactate levels and nutrient depletion, which can be reinforced through epigenetic modifications and intrinsic mutations. This process establishes a self-reinforcing metabolic loop that supports cancer progression.

Taken together, the metabolic characteristics of the immunosuppressive microenvironment in PDAC include elevated lactate concentrations, hypoxia, and a deficiency of metabolic substrates. The buildup of high lactate levels results in a reduction in both the quantity and functionality of antigen-presenting dendritic cells (DCs) (126). Furthermore, the differentiation and maturation of DCs can be inhibited by IL-6 and granulocyte-colony stimulating factor (G-CSF) released by cancer cells (127). Lactate suppresses nuclear factor of activated T cells (NFAT) in both T cells and NK cells, thereby reducing IFN-γ production and weakening antitumor immunosurveillance. Additionally, high lactate levels impair glucose transporter 10 (GLUT10), a key mediator of glucose uptake in CD8+ T cells. This metabolic disruption affects the PI3K-mechanistic target of rapamycin (mTOR) signaling axis, ultimately diminishing CD8^+^ T cell proliferation and antitumor function (128). Krol et al. (129) revealed that lactate promotes histone lactylation in Th17 cells, suppresses IL-17A expression, and drives their transdifferentiation into forkhead box P3 (Foxp3)-expressing Treg cells (129). Treg cells can enhance monocarboxylate transporter 1 (MCT1) expression, promoting lactate uptake and its subsequent conversion to phosphoenolpyruvate (PEP) through gluconeogenesis. PEP then enters glycolysis in a reversed flux, replenishing metabolic intermediates to support tumor cell proliferation (130). Lactate induces nuclear translocation of NFAT1, thereby upregulating PD-1 expression in Treg cells (131). Moreover, lactate upregulates the deubiquitinase ubiquitin-specific peptidase 39, facilitating CTLA-4 RNA splicing and ultimately enhancing CTLA-4 expression in Treg cells (132).The upregulation of these immune checkpoint molecules further suppresses immune responses. Under nutrient-deprived conditions, Treg cells undergo metabolic reprogramming by elevating fatty acid metabolism-related genes while suppressing glucose metabolism-associated genes to promote survival. Mechanistically, FOXP3 enhances fatty acid uptake in Tregs by transcriptionally upregulating the oxidized lipid scavenger receptor CD36 (133). In CD8^+^ T cells, elevated CD36 expression promotes excessive uptake of oxidized low-density lipoprotein, inducing LPO and p38 kinase activation, ultimately compromising T-cell functionality (134).

Metabolic changes in the microenvironment also recruit immunosuppressive cells and influence macrophage phenotypes. Macrophages can uptake lactate through MCT1–4 mediated by HiF-1α, which induces macrophages to secrete VEGF and Arginase 1 (Arg1)and differentiate into M2-like phenotype (135). Sun et al. (136) demonstrated that Kla of the non-histone protein α-endosulfine promotes STAT3 activation, triggering tumor cells to release CCL2. This chemokine recruits M2 macrophages, facilitating an immunosuppressive tumor microenvironment (136). Macrophages display elevated aryl hydrocarbon receptor (AhR) activity. Under inflammatory conditions, microenvironmental NO upregulates the transcription factor RUNT-related transcription factor 3 (RUNX3). RUNX3 binds to the indoleamine 2,3-dioxygenase 1 promoter, inducing its expression and catalyzing tryptophan breakdown into kynurenine (Kyn). Kyn then activates AhR, driving tumor progression and M2 macrophage polarization (137, 138). A recent study demonstrated that IL-4 drives the accumulation of 25-hydroxycholesterol (25HC) in the microenvironment, promoting M2 macrophage polarization. Lysosomal 25HC competitively binds the G protein-coupled receptor GPR155, suppressing mechanistic mTOR complex 1 activation. This suppression triggers STAT3 phosphorylation, elevating the production of M2-associated mediators—including Arg1 and IL-10—and ultimately reprograms macrophage function (139).

### 7.3 Cancer cells, CAFs, and the tumor immunosuppressive microenvironment

In PDAC, the immunosuppressive tumor microenvironment is centrally orchestrated by CAFs enables active recruitment and polarization of immunosuppressive myeloid populations—including MDSCs and M2-polarized TAMs. Concurrently, physical barriers and metabolic barriers are established to inhibit effector CD8^+^ T-cell trafficking. This CAF-dominated immunosuppressive niche consequently promotes tumor progression and systemic immune evasion. We next delineate the tripartite interplay among CAFs, carcinoma cells, and tumor-infiltrating immune cells, focusing on their integrated signaling networks within TME. iCAF subset serves as a critical molecular orchestrator of the immunosuppressive tumor microenvironment. iCAF activation is driven by IL-1α, IL-1β, TNF and STAT3 signaling pathways originating from neoplastic cells. As previously noted, IL-1β plays a role in the inflammatory interaction between cancer cells and macrophages (106, 140, 141). iCAFs and carcinoma cells cooperatively secrete chemokines and cytokines—including CCL2, CXCL1, IL-6, and GM-CSF to recruit circulating monocytes. These monocytes subsequently differentiate into TAMs and MDSCs within the tumor microenvironment (142–144). Numerous studies have established the role of tumor sialylation in immune regulation. A pivotal investigation by Kelly et al. (145) revealed that CAFs likewise generate sialic acids that engage immunosuppressive receptors Siglec-7, -9, -10, and -15 (sialic acid-binding immunoglobulin-type lectins). This interaction drives monocyte differentiation into CD163^+^CD206^+^ macrophages and impedes T cell proliferation. Notably, the sialyltransferase ST3 β-galactoside α-2,3-sialyltransferase 4, EC 2.4.99.4(ST3GAL4) was found to be overexpressed in CAFs. ST3GAL4 further contributes to synthesizing Siglec-9 ligands on PDAC cells, correlating with reduced survival in multivariate analysis. TAMs reciprocally influence CAFs, enhancing desmoplastic stroma formation. The seminal study by Lee et al. (146) demonstrated that macrophages express Oncostatin M (OSM), which engages the OSM receptor on CAFs. This ligand-receptor interaction potently induces overexpression of inflammatory genes characteristic of the iCAF phenotype and enriches protumoral pathways, including: KRAS signaling,IL-6/JAK/STAT3 signaling, PI3K/mTOR pathway and EMT (146). neutrophil extracellular traps induces dormant cancer cells to enter a proliferative state and increases the risk of lung metastasis (147). amyloid β protein secreted by CAFs binds to the CD11b receptor on neutrophils, driving their activation and facilitating the formation of this transition (148). CAFs drive pathological accumulation of ECM components. Matrix mechanical properties play dual roles in macrophage polarization: low matrix stiffness drives macrophages toward an M1 phenotype, whereas medium stiffness favors the transition to an M2 phenotype, highlighting the complexity of ECM mechanics in shaping the immune landscape (149).

CAFs can subvert antitumor immunity by hijacking T cell functions. Preclinical models have shown that CAFs secrete CXCL12, forming a protective coating around cancer cells, which identify T cells expressing CXCR4, repels T cells and prevents their infiltration into tumor tissues (150). In addition, dense matrix environment can substantially exacerbate T-cell exhaustion and impair antitumor immunity (151, 152). As previously described, the apCAF subset can induce CD4^+^ T cell differentiation into Tregs. However, emerging evidence indicates that apCAFs exhibit paradoxical functions beyond immunosuppression. Through integrative analysis of >14 million cells across 10 cancer types including PDAC on seven spatial transcriptomic and proteomic platforms, Liu et al. (153) revealed spatial heterogeneity among CAFs: apCAF-like subpopulations predominantly localize near tertiary lymphoid structures. These apCAFs highly express CXCL12 and CCL19 to sustain lymphocyte homing, correlating with favorable prognosis. Conversely, CAFs adjacent to tumor nests expressing TGF-β1, actin alpha 2 and IL-8 are enveloped by cellular microdomains richly populated with exhausted T cells and Tregs (153). This subset likely mediates immunosuppression via the TSP1-CD47 axis, generating dense stromal barriers that impede plasma cell infiltration (153).These findings demonstrate spatially stratified CAF functionalities in shaping PDAC immune landscapes. Furthermore, recent studies indicate that senescent CAF subpopulations restrict CD8^+^ T cell abundance and effector function. Combinatorial therapy with ICI and ABT-199 (Bcl-2 inhibitor) reshapes the TME, potentially reinvigorating antitumor immunity (154).

CAFs regulate immune cells through both direct and indirect mechanisms by modulating stromal components. Sensory neurons upregulate calcitonin gene-related peptide (CGRP) expression via NGF derived from CAFs. Upon binding to the receptor activity-modifying protein 1 on CAFs, CGRP suppresses IL-15 secretion, impairing NK cell antitumor function (155). Additionally, tumor-derived SHH engages PTCH1 on CAFs, activating GLI1-mediated transcriptional repression of anti-angiogenic factors thrombospondin-2 and TIMP2, thereby promoting neovascularization (156). In addition, macrophages can also promote neovascularization. Yang et al. (157) demonstrated a positive correlation between M2 macrophages and microvessel density in PDAC tissues. Exosomes produced by M2 macrophages contain miR-155-5p and miR-221-5p, which are transferred to endothelial cells and stimulate angiogenesis by selectively targeting and downregulating the transcription factor, E2 Factor 2.

### 7.4 Immunosuppression in cancer metastasis

PDAC is inherently a low-immunogenic tumor, which allows for immune evasion and metastasis through multiple routes, including hematogenous, lymphatic, and perineural pathways. The process of metastasis depends on the intrinsic characteristics of the primary tumor and the formation of a supportive premetastatic niche at distant sites (158). The liver is the most common site of PDAC metastasis, followed by the peritoneum and lungs (159). In murine models, the formation of a pre-metastatic niche in the liver is not solely due to anatomical proximity. However, it involves hepatocyte-mediated activation of the STAT3 pathway through IL-6 signaling, leading to serum amyloid A production and facilitating PDAC cell colonization (160).

Distinct metastatic sites exhibit unique microenvironmental characteristics. For example, lung metastases are marked by an increased infiltration of immune cells, including CD4^+^ T and CD8^+^ T cells, Tregs, dendritic cells, and macrophages. In contrast, liver metastases demonstrate low T cell infiltration, which may be linked to immunosuppressive pathways such as LAG3-FGL1 and secrete chemokine CXCL12-CXCR4 signaling (161). Recent studies have shown that PDAC cells can secrete mesothelin, which activates macrophages to produce VEGFα and S100A9. S100A9 enhances neutrophil recruitment and the formation of neutrophil extracellular traps, thereby promoting PDAC cell migration to the lungs (162).

Interactions between tumor cells and macrophages are critical to establish metastatic lesions. Modulating macrophage polarization represents a promising therapeutic strategy for mitigating metastasis. Stacy et al. (163) found that Kupffer cells, the resident macrophages of the liver, are potential targets for immunomodulation. Activation of Kupffer cells with β-glucan polarizes them towards an antigen-presenting phenotype, enhancing CD8^+^ T cell activation and rendering liver metastatic PDAC mice sensitive to anti-Programmed cell Death-1 therapy. This provides a new approach for immunotherapy to overcome immune tolerance in patients with advanced PDAC (163).

In summary, the interplay between cancer cells and immune cells is established early during tumorigenesis and persists through metastatic dissemination. Understanding the dynamic changes in the TME throughout PDAC progression is essential to identify novel therapeutic targets. In previous studies, neither single-agent immune checkpoint inhibitors nor dual-antibodies failed to achieve clinical benefit in PDAC (164). The current direction of immunotherapy for PDAC is to activate immunity and improve immunogenicity, such as CD40 activators, vaccines, CAR-T and TCR-T targeting specific antigens, and ADC drugs, and inhibit the recruitment of immunosuppressive cells, such as CXCR2 inhibitors and CSF1R inhibitors. Considering the mechanism of action between cancer cells and immune cells, we cannot limit ourselves to only a single-agent for immunization. We need to expand the direction of drug combination therapy. Clinical trials of multiple immune single-agent and combination therapies are underway.

## 8 Soluble pattern recognition receptors

Soluble Pattern Recognition Receptors (sPRRs) represent a category of non-transmembrane immune recognition molecules found in extracellular fluids, including plasma, tissue fluid, and mucosal secretions. These receptors initiate and regulate innate immune responses by detecting conserved pathogen- or damage-associated molecular patterns. Surfactant protein D (SP-D) fine-tunes cytokine and chemokine production at mucosal surfaces during infection, allergic reactions, and inflammatory processes. Research indicates that the recombinant fragment of human SP-D (rfhSP-D) can promote the upregulation of Fas, a pro-apoptotic marker in PDAC, subsequently initiating the caspase cascade to induce cell death (165). Furthermore, rfhSP-D can inhibit the EMT in pancreatic cancer by reducing TGF-β expression in PDAC and downregulating mesenchymal markers including Vimentin, Zeb1, and Snail (166). Not all soluble molecules contribute to anti-tumor immunity; some may have opposing effects. Yang et al. (167) conducted exome sequencing and RNA sequencing on primary tumors and paired liver metastases resected synchronously. Their findings indicate that tumors at the primary site can “educate” M2-type macrophages to secrete the pro-metastatic factor C1q, which plays a role in the formation of metastatic niches (167). Research indicates that tumor cells exhibit overexpression of globular C1q receptor, facilitating its binding to the globular head of plasma C1q. This interaction inhibits C1q from binding to immune complexes and initiating complement activation, thus promoting tumor immune evasion (168). Malassezia is significantly enriched in cancer tissues compared to normal tissues, and bacterial dysbiosis has also been linked to the carcinogenic process of PDAC. The glycan in the fungal wall binds to mannose-binding lectin in the C-type lectin superfamily, activating the complement cascade and accelerating the progression of cancer (169).

## 9 Exosomes and the microenvironment: signaling mediators

Exosomes are nanoscale (50–150 nm) extracellular vesicles(EVs)generated through inward budding of endosomal membranes, forming intraluminal vesicles within multivesicular endosomes (MVEs)—key intermediates in the endosomal trafficking pathway. They are released upon MVE-plasma membrane fusion and transport a diverse cargo of proteins, nucleic acids, and lipids. In various cancers, exosomes facilitate intercellular crosstalk and play crucial roles in immunomodulation (170, 171). Exosomes serve as bidirectional “instructive messengers” between cancer cells and microenvironmental components. They mediate immune evasion and facilitate crosstalk among tumor cells, stromal cells, and immune cells. Multiple studies demonstrate that cancer cell-derived exosomes carry coding RNAs, proteins, and metabolites that suppress immune responses and promote tumor-promoting phenotypes. These exosomes are enriched with diverse immunosuppressive molecules (including PD-L1, FasL, TRAIL, and CTLA-4), major histocompatibility complexes (MHC-I/II), immunoregulatory cytokines (IL-10, TGF-β and PGE2), and ectoenzymes involved in the adenosine pathway (CD39 and CD73). These components collectively activate or suppress immune cells within the TME (172, 173). For example, a study by Zhou et al. (173) revealed that cancer cell-derived exosomal microRNA-203 downregulates TLR4 in immature DCs and associated cytokines –TNFα and IL-12. Furthermore, tumor-derived exosomal heat shock protein 72 enhances MDSC expansion by activating STAT3 via TLR2/MyD88-dependent autocrine IL-6 production, reinforcing an immunosuppressive tumor microenvironment TME. Additionally, exosomes modulate macrophage polarization under oxidative stress. Specifically, exosomally delivered KRAS^G12D^ triggers STAT3 signaling in macrophages through the receptor for advanced glycation end products, upregulating fatty acid oxidation associated factors and driving M2-like macrophage polarization (174). Notably, Wang et al. (175) revealed that PDAC-derived small EV-carried microRNA-301a-3p promotes M2 macrophage polarization via the phosphatase and TENsin homolog (PTEN)/PI3Kγ axis, dependent on HIF-1α or HIF-2α under hypoxia (175). Studies demonstrate that tumor-derived EVs are pivotal in modulating the tumor TME to facilitate metastatic niche formation. Exosomal CD44 engages integrin α6β4 on hepatocytes, triggering downstream cascades (e.g., c-Src/Ras pathways) to enhance migration. Additionally, it activates MMP-9—promoting ECM degradation—and remodels the ECM via hyaluronic acid binding. These interactions collectively drive cancer cell colonization and liver premetastatic niche formation (176). CD44v6 is an exosome-derived biomarker of pancreatic cancer-initiating cells and cancer stem cells, contributing to tumor motility and invasiveness. Separately, complement component 1q binding protein (C1QBP), a C1q receptor, critically regulates inflammatory responses. A study by Xie et al. (177) revealed that the exosomal CD44v6/C1QBP complex is internalized by hepatic stellate cells (HSCs), activating the insulin-like growth factor-1 signaling pathway to induce a fibrotic microenvironment that facilitates liver metastasis. Additionally, exosomal tRF-Glu-CTC-0005 activates HSCs by binding WDR1 mRNA, blocking its degradation and upregulating WDR1 protein—an actin depolymerization regulator—to drive liver metastasis (178).

Notably, multiple stromal constituents within the microenvironment secrete exosomes that mediate tumor invasion, immune evasion, and chemoresistance. Non-coding RNAs in PSC derived exosomes serve as critical mediators driving PDAC progression. Cao et al. (179) identified exosomal tRF-19-PNR8YPJZ from PSCs, which, upon transfer to PDAC cells, activates the Wnt/β-catenin pathway by binding and stabilizing AXIN2—a critical regulator of β-catenin turnover—thereby enhancing tumor proliferation and migration (179). Furthermore, exosomal miR-5703 from PSCs binds to CKLF-like MARVEL transmembrane domain-containing 4 in PDAC cells, inducing G2/M arrest while simultaneously promoting proliferation via p21-activated kinase 4-mediated activation of the PI3K/Akt pathway (180). Additionally, PSC-derived exosomes loaded with lncRNA UCA1, secreted protein acidic and rich in cysteine, CXCL12, and immunosuppressive molecules drive GEM resistance in PDAC (181, 182). CAF-secreted miRNAs further contribute to PDAC chemoresistance. For instance, exosomal miR-3173-5p inhibits GEM-induced ferroptosis by blocking acyl-CoA synthetase long-chain family member 4 (a key ferroptosis regulator via fatty acid metabolism activation). Strikingly, these findings oppose the traditional view of intrinsic CAF resistance to GEM, suggesting instead that PDAC chemoresistance arises from cooperative CAF-cancer cell crosstalk (183).Furthermore, exosomal microRNA-92a promotes chemoresistance by inducing degradation of phosphatase and tensin homolog mRNA(a key tumor suppressor) (184). Yao et al. (185) revealed that tumor-derived exosomal lncRNA RP11-161H23.5 interacts with a CCR4-NOT deadenylase complex subunit to attenuate HLA-A expression. This mechanism impairs CD8^+^ T-cell function by diminishing IFN-γ, TNF-α, and granzyme B production, thereby promoting immune escape and conferring immunotherapy resistance. Exosomes derived from M2-polarized macrophages play a critical role in promoting tumor progression. Ubiquitination critically regulates tumorigenesis by modulating cell survival, proliferation, and differentiation (186). Recent studies show that M2 macrophage-derived exosomal miR-193b-3p promotes tumor proliferation, migration, invasion, and glutamine uptake by suppressing tripartite motif-containing protein 6 (an E3 ligase) and stabilizing c-Myc via impaired ubiquitination (187). M2 macrophage-derived exosomal miR-501-3p promotes PDAC progression by activating TGF-β signaling and suppressing transforming growth factor beta receptor 3, a tumor suppressor (188).

## 10 Treatment

**Figure 2:**
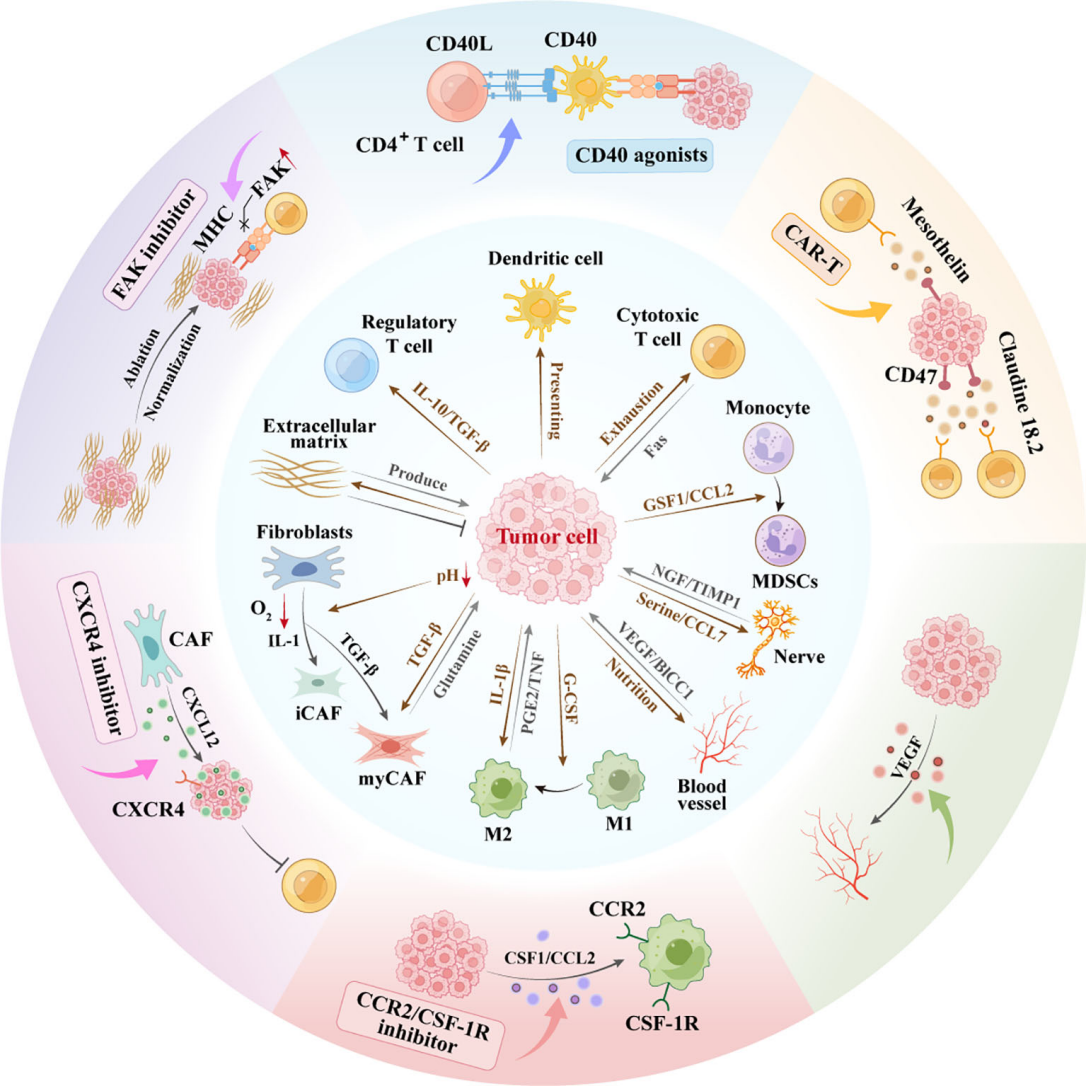
Interaction network between pancreatic cancer cells and microenvironment. Schematic representation of the complex interactions within the PDAC and TME. CAF Heterogeneity: PDAC cells secrete TGF-β inducing CAF differentiation. Hypoxia/IL-1 drive iCAFs; TGF-β forms myCAFs, differentially regulating progression. ECM Duality: Cancer/CAF-derived ECM supports growth yet impedes treatment via physical barriers. Angiogenesis:Pro-angiogenic factors from PDAC drive neovascularization, fueling growth/metastasis. Perineural Invasion: TME nerves secrete tryptophan/CCL7 stimulating proliferation; PDAC cells secrete NGF enabling neural invasion.Immunosuppression: PDAC recruits TAMs/MDSCs via CSF1/CCL2/IL-10, establishing immune-evasive microenvironments. CAR-T, chimeric antigen receptor T-cell; FAK, focal adhesion kinase; MDSC, myeloid-derivedsuppressorcell; M1, M1-Polarized Macrophages; M2, M2-Polarized Macrophages; VEGF, vascular endothelial growth factor.

Given the intricate crosstalk between PDAC cells and the tumor microenvironment TME, therapeutic strategies must target both cancer cells and their tumor-supporting stroma, either by disrupting protumorigenic interactions or through combination approaches. Notably, combination therapies synergistically disrupt PDAC-TME crosstalk, remodel the immunosuppressive microenvironment, and potentiate immunotherapy response. However, a critical challenge lies in the precise identification of actionable targets within the TME, given its complexity and heterogeneity (**Figure 2**).

With the integration of single-cell and multi-omics technologies in the analysis of the pancreatic cancer microenvironment, novel therapeutic strategies targeting the most prominent stromal component, CAFs, have been proposed. Current strategies include inhibiting key signaling pathways such as the CXCL12-CXCR4 axis (150), suppression of FAK (189), and blockade of the JAK-STAT pathway. In addition, ECM degradation through targeting CAF-mediated ECM deposition has shown promise (190–192). Emerging approaches, such as MesoFAP CAR-T cell therapy (193), autophagy inhibition (194), and CAF reprogramming, have also been explored. The modulation of ECM properties through normalization, remodeling, or stromal softening (195) has yielded promising results in preclinical models, and several of these approaches are progressing to early-stage clinical trials. Key strategies targeting pancreatic cancer’s immunosuppressive TME include: (1) BTK inhibitors to suppress regulatory B cells (196, 197), and CC2R and GSF-1R inhibitors targeting Immunosuppressive cells (198). Activating the innate immunity includes CD40 agonists that enhance antigen presentation, CAR-T cells targeting cancer cell-specific antigens (such as CEA CAR-T), and bispecific T-cell engager antibody therapies (199), Cancer vaccines (such as those targeting immune checkpoints CD47, CD73 and Claudin 18.2/CLDN18.2) and antibody-drug conjugate drugs. As described above regarding the immunosuppressive microenvironment, preclinical studies have shown promising results from combining *KRAS* mutations with immunotherapy. And currently, relevant Phase I clinical trials are ongoing for recruitment. In addition, targeting epigenetics and metabolism in combination with immunotherapy will also be a therapeutic strategy worthy of exploration in clinical trials.

Biochemical technologies have emerged as critical adjuncts in cancer therapy. For instance, liposomal nanoparticle delivery systems exhibit enhanced cellular permeability and improved bioavailability, thereby optimizing therapeutic efficacy and reducing off-target effects (200). For example, a polymer micelle-based nanomedicine (named M-CPA/PTX) for co-delivery of SHH inhibitor and paclitaxel has significantly prolonged the survival of mice (201). vaccines targeting immune checkpoints (CD47, CD73) and Claudin 18.2 (CLDN18.2) Similarly, vascular-targeted photodynamic therapy (202) is being investigated as a promising strategy for selective tumor ablation. However, caution must be exercised when translating preclinical findings into clinical applications, as TME complexities differ significantly between murine models and humans. Consequently, therapies targeting a single molecular pathway may fail to capture the multifaceted nature of TME. Moreover, traditional clinical trial designs, which often rely on broad patient stratification, may not adequately account for the heterogeneity of patient responses. Instead, precision oncology approaches, such as umbrella trials (203)–which stratify patients based on predictive biomarkers and other risk factors–may be more conducive to the current precise combination therapy. **Tables 3**, **4** summarize completed and ongoing clinical trials exploring combination therapies targeting neoplastic cells and their microenvironmental components.

**Table 3:** Some clinical trials targeting tumor cells and components of the TME in PDAC.

Therapeutic mechanism	ID	Phase	Patient population	Targeting cancer tissue	ICB	Targeting microenvironment	Preclinical rationale	Significant result
Targeting stromal and tumor elements	NCT05669482	Phase Ib/II	Untreated metastatic pancreatic cancers	AG Avutometinib (RAF/MEK clamp)	NA	Defactinib (FAK)	↓Fibrosis ↑Antigen-presenting ↑CD8^+^T cells (189, 204)	DCR is 100%; 6/8 patients are PR
	NCT02826486	Phase II	Previously treated metastatic PDAC	NanoliposomalIrinotecan, Fluorouracil, and Folinic acid	Pembrolizumab	Motixafortide (CXCR4)	↑CD8 ^+^Tcells infiltration ↓CAFs ↑PD-1 ICI effect (150)	ORR 21.1%
	NCT02715804	Phase III	Untreated metastatic and hyaluronan-high pancreatic cancers	AG	NA	PEGPH20	↓ECM (29)↓ECM (29)	No OS or PFS improved
	NCT01959139	Phase Ib/II	Untreated metastatic pancreatic cancers	mFOLFIRINOX	NA	PEGPH20	↓ECM	Compared with mFOLFIRINOX alone, toxicity increased, and the mOS shorten
	NCT02545504	Phase I	Advanced pancreatic adenocarcinoma	AG	NA	GS‐5745 (MMP9)	↓ECM ↑Reshaping the matrix ↓EMT (205, 206)	PFS 7.8 months PFS is 7.8 months; 16 PRs (n=36)
	NCT01088815	Phase II	Patients with previously untreated metastatic pancreatic adenocarcinoma	AG	NA	Vismodegib (The Hedgehog signaling pathway)	↓ECM ↓myCAFs↑iCAFs (29, 54)↓myCAFs↑iCAFs (29, 54)	No improved efficacy
	NCT02117479	Phase III	Metastatic PDAC after disease progression	Capecitabine	NA	Ruxolitinib (JAK-STAT)	↓iCAFs ↑myCAFs (207, 208)	No improved survival
Targeting blood vessels and tumor elements	NCT00088894	Phase III	Advanced PDAC	Gemcitabine	NA	Bevacizumab	↓Angiogenesis↑vascular normalization (71, 81)↓Angiogenesis↑vascular normalization (71, 81)	No OS or PFS improved
	NCT02581215	Phase II	Advanced PDAC	mFOLFIRINOX	NA	Ramucirumab	↓Angiogenesis ↑vascular normalization (209, 210)	No OS or PFS improved
	NCT05493995	Phase II	Metastatic PDAC	AG	Penpulimab	Anlotinib (TKI inhibitor)	↓Multiple pro-angiogenetic signaling pathways (211, 212)	ORR is 50% (33/66); mOS is 13.7 months
	ChiCTR2000030659	Phase II	Patients with PDAC liver metastasis who have received first-line treatment	S-1	Sintilimab	Anlotinib	↓Multiple pro-angiogenetic signaling pathways	The ORR was 10.5% (95% CI 0.4–25.7%) in the evaluable population
Targeting immune suppressive cells and tumor elements	NCT02436668	Phase III	Metastatic pancreatic adenocarcinoma	AG	NA	Ibrutinib (BTK inhibitor)	↓CD1dhiCD5 ^+^Breg ↓PanIN growth ↑M1 (196, 197)	No OS or PFS improved
	NCT02562898	Phase Ib	Patients with advanced PDAC	NA	NA	Ibrutinib	↓CD1dhiCD5 ^+^Breg ↓PanIN growth ↑M1	In the circulation and TME, T cells, monocytes and DCs increased
	NCT02732938	Phase Ib	Metastatic PDAC	AG	NA	PF-04136309 (CCR2)	↑Reprogramming of TAM ↑increased T-cell infiltration (198)	No efficiency
	NCT02880371	Phase Ib/II	Advanced Solid Tumors	NA	Pembrolizumab	ARRY-382 (GSF-1R)	↓M2 ↑M1 ↑T cell infiltration (213)	1/27 patients had a PR lasting 2.4 months
	NCT01413022	Phase Ib	Patients with borderline resectable and locally advanced PDAC	FOLFIRINOX	NA	PF-04136309 (CCR2)	↓M2 ↑M1 ↑T cell infiltration (213)	Of 33 patients, 16 (49%) achieved ORR after repeat imaging.
Vaccine	NCT02451982	Phase II	Resectable pancreatic adenocarcinoma	Cyclophosphamide	Nivolumab	GVAX Urelumab (anti-CD137 agonist)	↑GM-CSF; ↑adaptive and innate immunity (144); ↑active T cell (214, 215)	mOS is 35.55 months
	NCT02243371	Phase II b	Previously treated metastatic PDAC	Cyclophosphamide	Nivolumab	GVAX (include cyclophosphamide) CRS-207 (mesothelin)	↑GM-CSF; ↑adaptive and innate immunity (144)	Not meet its primary efficacy endpoint
	NCT04161755	Phase I	Postoperative PDAC	NA	NA	Cevumeran (mRNA)	↑T cell immune (216)	8/16 patients had a longer median recurrence-free survival
	NCT02854072	Phase III	Previously untreated patients with PDAChaving high serum eotaxin levels	Gemcitabine/capecitabine	NA	GV1001 (hTERT)	↑Specific T-cell responses; ↑ Long-term T-cell memory (217)	mOS is 11.3 months; TTP is 7.3 months
Targeting CD40	NCT03214250	Phase II	Metastatic PDAC	AG	Nivolumab	Sotigalimab	↑DC activity ↑CD8^+^Tcells (218, 219)	No difference from historical 1-year OS (35%)
	NCT04888312	phase Ib/II	Previously untreated metastatic PDAC	mFOLFIRINOX	NA	Mitazalimab	↑DC activity ↑CD8^+^T cells	ORR is 40.4%, mOS is 14.3 months

CR, complete remission; ORR, overall remission rate; PFS, progression-free survival; OS, overall survival; DCR, disease control rate; RFS, recurrence free survival; TTP, time to progression; mFOLFIRINOX, modified oxaliplatin, irinotecan, fluorouracil, and calcium folinate; PR, partial remission; S-1, Tegafur; ↑, denotes upregulation or increase;↓, denotes downregulation or decrease.

**Table 4:** The ongoing new direction clinical trials targeting tumor cells and components of the TME in PDAC.

ID	Phase	Patient population	Type of Therapy
NCT05827796	phase Ib/II	Advanced pancreatic cancer	IN10018(FAK inhibitor) plus KN046(the anti-PD-L1/CTLA-4 bispecific antibody) plus AG
NCT05355298	Phase Ib/IIa	Unresectable or metastatic pancreatic cancer.	AMP945(FAK inhibitor) plus AG
NCT06182072	Phase I/Ib	Previously untreated metastatic PDAC	ProAgio(anti- αvβ3 integrin cytotoxin) plus AG
NCT05077800	Phase II	Previously untreated metastatic PDAC	FOLFIRINOX plus 9-ING-41(glycogen synthase kinase-3 beta inhibitor)and losartan(TGF-β inhibitor)
NCT06141031	Phase I/II	Borderline resectable pancreatic cancer	Radiotherapy plus TTI-101 (inhibitor of STAT3)
NCT04803851	PhaseI/II	Advanced pancreatic cancer	Anlotinib(TKI inhibitor) plus AK105(anti-PD-1 antibody)
NCT05481476	Phase II	Locally advanced or metastatic pancreatic cancer	Surufatinib plus Sintilimab plus AG
NCT05481463	Phase II	Advanced pancreatic cancer	Surufatinib plus TAS-102
NCT05919238	Phase I	Locally advanced unresectable PDAC	Padeliporfin VTP
NCT06119217	Phase II	Metastatic pancreatic adenocarcinoma	TTX-030 (a anti-CD39 antibody) plus Budigalimab plus AG
NCT04524702	Phase II	Advanced pancreatic cancer	Paricalcitol(vitamin D receptor agonists) plus Hydroxychloroquine(autophagic flux inhibitor) plus AG
NCT04669197	Phase II	Untreated resectable, borderline resectable and locally advanced adenocarcinoma of the pancreas	AG plus Cisplatin plus Hydroxychlororoquine
NCT05482893	Phase 1/2	Unresectable or metastatic gastric adenocarcinoma, gastroesophageal junction adenocarcinoma and PDAC	PT886(anti-claudin18.2/anti-CD47 bispecific antibody)plus chemotherapy/pembrolizumab
NCT04940286	Phase II	Resectable/​Borderline Resectable primary pancreatic cancer	AG plus Durvalumab plus Oleclumab9(an anti-CD73 antibody)
NCT05431270	Phase I	Locally advanced or metastatic solid tumors	PT199 (an Anti-CD73 antibody) plus PD-1 Inhibitor
NCT06496373	Phase I	Postoperative PDAC	XP-004 personalized mRNA tumor vaccine plus PD-1 inhibitor
NCT05916261	Phase I	Advanced pancreatic cancer	Personalized tumor vaccines mRNA-0217/S001 plus Pabolizumab
NCT05721846	Phase I	Refractory pancreatic cancer	Nivolumab plus Ipilimumab plus TGFβ-15 peptide vaccine plus Stereotactic body Radiotherapy
NCT06205849	Phase I	Locally advanced pancreas cancer	Intra-tumoral mitazalimab plus Irreversible electroporation
NCT05438667	Phase I	Advanced pancreatic cancer	KRAS mutant antigen specific TCR-T cells
NCT05779917	Phase I	advanced pancreatic cancer	Mesothelin/​GPC3/​GUCY2C-CAR-T Cells

## 11 Conclusion and future directions

The PDAC microenvironment exhibits distinct characteristics, such as low immunogenicity, desmoplastic stroma, hypovascularity, and an immunosuppressive landscape, collectively contributing to its therapeutic resistance. These unique TME features distinguish PDAC from other solid malignancies, thereby complicating treatment efficacy and limiting therapeutic response. Thus, elucidating the complex interplay between tumor cells and their surrounding stroma is crucial for devising more effective therapeutic strategies.

Recent advancements, including multi-omics profiling and single-cell sequencing, have significantly enhanced our understanding of the intricate cellular and molecular interactions within the PDAC microenvironment. These state-of-the-art techniques have facilitated the identification of key cellular subpopulations, signaling pathways, and stromal components that regulate tumor progression and treatment resistance, leading to the identification of novel therapeutic targets and facilitating the development of precision medicine approaches.

In this review, we comprehensively delineate the latest progress in understanding how tumor cells manipulate and exploit the TME to sustain their growth and evade immune surveillance. Given the interplay between various cellular and acellular components within the PDAC stroma, a multifaceted therapeutic approach targeting the network of TME interactions is essential to overcome the limitations of monotherapies. We speculate that a deeper understanding of the unique PDAC microenvironmental dynamics will enable the rational design of combination therapies that can disrupt these protumorigenic interactions. By integrating these insights into clinical practice, we can develop precision-based treatment strategies tailored to the specific TME profiles of patients with PDAC, improving therapeutic outcomes and mitigating resistance.

## References

[1] Siegel RL Giaquinto AN Jemal A. Cancer statistics, 2024. CA Cancer J Clin. (2024) 74:12–49. doi: 10.3322/caac.21820, PMID: 38230766

[2] Rahib L Wehner MR Matrisian LM Nead KT. Estimated projection of US cancer incidence and death to 2040. JAMA Netw Open. (2021) 4:e214708. doi: 10.1001/jamanetworkopen.2021.4708, PMID: 33825840 PMC8027914

[3] Wang J Yang J Narang A He J Wolfgang C Li K. Consensus, debate, and prospective on pancreatic cancer treatments. J Hematol Oncol. (2024) 17:92. doi: 10.1186/s13045-024-01613-x, PMID: 39390609 PMC11468220

[4] Strobel O Neoptolemos J Jäger D Büchler MW. Optimizing the outcomes of pancreatic cancer surgery. Nat Rev Clin Oncol. (2019) 16:11–26. doi: 10.1038/s41571-018-0112-1, PMID: 30341417

[5] Grossberg AJ Chu LC Deig CR Fishman EK Hwang WL Maitra A. Multidisciplinary standards of care and recent progress in pancreatic ductal adenocarcinoma. CA Cancer J Clin. (2020) 70:375–403. doi: 10.3322/caac.21626, PMID: 32683683 PMC7722002

[6] Rompen IF Levine J Habib JR Sereni E Mughal N Hewitt DB. Progression of site-specific recurrence of pancreatic cancer and implications for treatment. Ann Surg. (2024) 280:317–24. doi: 10.1097/SLA.0000000000006142, PMID: 37870253 PMC11259998

[7] Park W Chawla A O’reilly EM. Pancreatic cancer: A review. JAMA. (2021) 326:851–62. doi: 10.1001/jama.2021.13027, PMID: 34547082 PMC9363152

[8] Goldstein D El-Maraghi RH Hammel P Heinemann V Kunzmann V Sastre J. nab-Paclitaxel plus gemcitabine for metastatic pancreatic cancer: long-term survival from a phase III trial. J Natl Cancer Inst. (2015) 107(2). doi: 10.1093/jnci/dju413, PMID: 25638248

[9] Brahmer JR Tykodi SS Chow LQ Hwu WJ Topalian SL Hwu P. Safety and activity of anti-PD-L1 antibody in patients with advanced cancer. N Engl J Med. (2012) 366:2455–65. doi: 10.1056/NEJMoa1200694, PMID: 22658128 PMC3563263

[10] Luo W Wen T Qu X. Tumor immune microenvironment-based therapies in pancreatic ductal adenocarcinoma: time to update the concept. J Exp Clin Cancer Res. (2024) 43:8. doi: 10.1186/s13046-023-02935-3, PMID: 38167055 PMC10759657

[11] De Visser KE Joyce JA. The evolving tumor microenvironment: From cancer initiation to metastatic outgrowth. Cancer Cell. (2023) 41:374–403. doi: 10.1016/j.ccell.2023.02.016, PMID: 36917948

[12] Collisson EA Sadanandam A Olson P Gibb WJ Truitt M Gu S. Subtypes of pancreatic ductal adenocarcinoma and their differing responses to therapy. Nat Med. (2011) 17:500–3. doi: 10.1038/nm.2344, PMID: 21460848 PMC3755490

[13] Moffitt RA Marayati R Flate EL Volmar KE Loeza SG Hoadley KA. Virtual microdissection identifies distinct tumor- and stroma-specific subtypes of pancreatic ductal adenocarcinoma. Nat Genet. (2015) 47:1168–78. doi: 10.1038/ng.3398, PMID: 26343385 PMC4912058

[14] Tu M Klein L Espinet E Georgomanolis T Wegwitz F Li X. TNF-α-producing macrophages determine subtype identity and prognosis via AP1 enhancer reprogramming in pancreatic cancer. Nat Cancer. (2021) 2:1185–203. doi: 10.1038/s43018-021-00258-w, PMID: 35122059

[15] Klein L Tu M Krebs N Urbach L Grimm D Latif MU. Spatial tumor immune heterogeneity facilitates subtype co-existence and therapy response in pancreatic cancer. Nat Commun. (2025) 16:335. doi: 10.1038/s41467-024-55330-7, PMID: 39762215 PMC11704331

[16] Puleo F Nicolle R Blum Y Cros J Marisa L Demetter P. Stratification of pancreatic ductal adenocarcinomas based on tumor and microenvironment features. Gastroenterology. (2018) 155:1999–2013.e3. doi: 10.1053/j.gastro.2018.08.033, PMID: 30165049

[17] Jiang D Guo R Machens HG Rinkevich Y. Diversity of fibroblasts and their roles in wound healing. Cold Spring Harb Perspect Biol. (2023) 15(3). doi: 10.1101/cshperspect.a041222, PMID: 36167647 PMC9979851

[18] Buechler MB Pradhan RN Krishnamurty AT Cox C Calviello AK Wang AW. Cross-tissue organization of the fibroblast lineage. Nature. (2021) 593:575–9. doi: 10.1038/s41586-021-03549-5, PMID: 33981032

[19] Chhabra Y Weeraratna AT. Fibroblasts in cancer: Unity in heterogeneity. Cell. (2023) 186:1580–609. doi: 10.1016/j.cell.2023.03.016, PMID: 37059066 PMC11422789

[20] Bagger MM Sjölund J Kim J Kohler KT Villadsen R Jafari A. Evidence of steady-state fibroblast subtypes in the normal human breast as cells-of-origin for perturbed-state fibroblasts in breast cancer. Breast Cancer Res. (2024) 26:11. doi: 10.1186/s13058-024-01763-3, PMID: 38229104 PMC10790388

[21] Matsumura K Hayashi H Uemura N Ogata Y Zhao L Sato H. Thrombospondin-1 overexpression stimulates loss of Smad4 and accelerates Malignant behavior via TGF-β signal activation in pancreatic ductal adenocarcinoma. Transl Oncol. (2022) 26:101533. doi: 10.1016/j.tranon.2022.101533, PMID: 36115074 PMC9483797

[22] Donahue KL Watkoske HR Kadiyala P Du W Brown K Scales MK. Oncogenic KRAS-dependent stromal interleukin-33 directs the pancreatic microenvironment to promote tumor growth. Cancer Discov. (2024) 14:1964–89. doi: 10.1158/2159-8290.CD-24-0100, PMID: 38958646 PMC11450371

[23] Parte S Kaur AB Nimmakayala RK Ogunleye A O Chirravuri R Vengoji R. Cancer-associated fibroblast induces acinar-to-ductal cell transdifferentiation and pancreatic cancer initiation via LAMA5/ITGA4 axis. Gastroenterology. (2024) 166:842–58.e5. doi: 10.1053/j.gastro.2023.12.018, PMID: 38154529 PMC11694316

[24] Storz P. Acinar cell plasticity and development of pancreatic ductal adenocarcinoma. Nat Rev Gastroenterol Hepatol. (2017) 14:296–304. doi: 10.1038/nrgastro.2017.12, PMID: 28270694 PMC6036907

[25] Von Ahrens D Bhagat TD Nagrath D Maitra A Verma A. The role of stromal cancer-associated fibroblasts in pancreatic cancer. J Hematol Oncol. (2017) 10:76. doi: 10.1186/s13045-017-0448-5, PMID: 28351381 PMC5371211

[26] Corcoran RB Contino G Deshpande V Tzatsos A Conrad C Benes CH. STAT3 plays a critical role in KRAS-induced pancreatic tumorigenesis. Cancer Res. (2011) 71:5020–9. doi: 10.1158/0008-5472.CAN-11-0908, PMID: 21586612 PMC3693754

[27] Rhim AD Oberstein PE Thomas DH Mirek ET Palermo CF Sastra SA. Stromal elements act to restrain, rather than support, pancreatic ductal adenocarcinoma. Cancer Cell. (2014) 25:735–47. doi: 10.1016/j.ccr.2014.04.021, PMID: 24856585 PMC4096698

[28] Caligiuri G Tuveson DA. Activated fibroblasts in cancer: Perspectives and challenges. Cancer Cell. (2023) 41:434–49. doi: 10.1016/j.ccell.2023.02.015, PMID: 36917949 PMC11022589

[29] Olive KP Jacobetz MA Davidson CJ Gopinathan A Mcintyre D Honess D. Inhibition of Hedgehog signaling enhances delivery of chemotherapy in a mouse model of pancreatic cancer. Science. (2009) 324:1457–61. doi: 10.1126/science.1171362, PMID: 19460966 PMC2998180

[30] Auciello FR Bulusu V Oon C Tait-Mulder J Berry M Bhattacharyya S. A stromal lysolipid-autotaxin signaling axis promotes pancreatic tumor progression. Cancer Discov. (2019) 9:617–27. doi: 10.1158/2159-8290.CD-18-1212, PMID: 30837243 PMC6497553

[31] Francescone R Barbosa Vendramini-Costa D Franco-Barraza J Wagner J Muir A Lau AN. Netrin G1 promotes pancreatic tumorigenesis through cancer-associated fibroblast-driven nutritional support and immunosuppression. Cancer Discov. (2021) 11:446–79. doi: 10.1158/2159-8290.CD-20-0775, PMID: 33127842 PMC7858242

[32] Murthy D Attri KS Shukla SK Thakur R Chaika NV He C. Cancer-associated fibroblast-derived acetate promotes pancreatic cancer development by altering polyamine metabolism via the ACSS2-SP1-SAT1 axis. Nat Cell Biol. (2024) 26:613–27. doi: 10.1038/s41556-024-01372-4, PMID: 38429478 PMC11021164

[33] Sahai E Astsaturov I Cukierman E Denardo DG Egeblad M Evans RM. A framework for advancing our understanding of cancer-associated fibroblasts. Nat Rev Cancer. (2020) 20:174–86. doi: 10.1038/s41568-019-0238-1, PMID: 31980749 PMC7046529

[34] Helms EJ Berry MW Chaw RC Dufort CC Sun D Onate MK. Mesenchymal lineage heterogeneity underlies nonredundant functions of pancreatic cancer-associated fibroblasts. Cancer Discov. (2022) 12:484–501. doi: 10.1158/2159-8290.CD-21-0601, PMID: 34548310 PMC8831457

[35] Zeisberg EM Potenta S Xie L Zeisberg M Kalluri R. Discovery of endothelial to mesenchymal transition as a source for carcinoma-associated fibroblasts. Cancer Res. (2007) 67:10123–8. doi: 10.1158/0008-5472.CAN-07-3127, PMID: 17974953

[36] Huang H Wang Z Zhang Y Pradhan RN Ganguly D Chandra R. Mesothelial cell-derived antigen-presenting cancer-associated fibroblasts induce expansion of regulatory T cells in pancreatic cancer. Cancer Cell. (2022) 40:656–73.e7. doi: 10.1016/j.ccell.2022.04.011, PMID: 35523176 PMC9197998

[37] Huang X He C Hua X Kan A Mao Y Sun S. Oxidative stress induces monocyte-to-myofibroblast transdifferentiation through p38 in pancreatic ductal adenocarcinoma. Clin Transl Med. (2020) 10:e41. doi: 10.1002/ctm2.41, PMID: 32508052 PMC7403727

[38] Iwamoto C Ohuchida K Shinkawa T Okuda S Otsubo Y Okumura T. Bone marrow-derived macrophages converted into cancer-associated fibroblast-like cells promote pancreatic cancer progression. Cancer Lett. (2021) 512:15–27. doi: 10.1016/j.canlet.2021.04.013, PMID: 33961925

[39] Miyazaki Y Oda T Mori N Kida YS. Adipose-derived mesenchymal stem cells differentiate into pancreatic cancer-associated fibroblasts *in vitro* . FEBS Open Bio. (2020) 10:2268–81. doi: 10.1002/2211-5463.12976, PMID: 32931156 PMC7609785

[40] Niu N Shen X Wang Z Chen Y Weng Y Yu F. Tumor cell-intrinsic epigenetic dysregulation shapes cancer-associated fibroblasts heterogeneity to metabolically support pancreatic cancer. Cancer Cell. (2024) 42:869–84.e9. doi: 10.1016/j.ccell.2024.03.005, PMID: 38579725

[41] Elyada E Bolisetty M Laise P Flynn WF Courtois ET Burkhart RA. Cross-species single-cell analysis of pancreatic ductal adenocarcinoma reveals antigen-presenting cancer-associated fibroblasts. Cancer Discov. (2019) 9:1102–23. doi: 10.1158/2159-8290.CD-19-0094, PMID: 31197017 PMC6727976

[42] Mucciolo G Araos Henríquez J Jihad M Pinto-Teles S Manansala JS Li W. EGFR-activated myofibroblasts promote metastasis of pancreatic cancer. Cancer Cell. (2024) 42:101–18.e11. doi: 10.1016/j.ccell.2023.12.002, PMID: 38157863

[43] Ge F Zeng C Wang J Liu X Zheng C Zhang H. Cancer-associated fibroblasts drive early pancreatic cancer cell invasion via the SOX4/MMP11 signalling axis. Biochim Biophys Acta Mol Basis Dis. (2024) 1870:166852. doi: 10.1016/j.bbadis.2023.166852, PMID: 37633471

[44] Öhlund D Handly-Santana A Biffi G Elyada E Almeida AS Ponz-Sarvise M. Distinct populations of inflammatory fibroblasts and myofibroblasts in pancreatic cancer. J Exp Med. (2017) 214:579–96. doi: 10.1084/jem.20162024, PMID: 28232471 PMC5339682

[45] Chen K Wang Q Li M Guo H Liu W Wang F. Single-cell RNA-seq reveals dynamic change in tumor microenvironment during pancreatic ductal adenocarcinoma Malignant progression. EBioMedicine. (2021) 66:103315. doi: 10.1016/j.ebiom.2021.103315, PMID: 33819739 PMC8047497

[46] Wang Y Liang Y Xu H Zhang X Mao T Cui J. Single-cell analysis of pancreatic ductal adenocarcinoma identifies a novel fibroblast subtype associated with poor prognosis but better immunotherapy response. Cell Discov. (2021) 7:36. doi: 10.1038/s41421-021-00271-4, PMID: 34035226 PMC8149399

[47] Mizutani Y Kobayashi H Iida T Asai N Masamune A Hara A. Meflin-positive cancer-associated fibroblasts inhibit pancreatic carcinogenesis. Cancer Res. (2019) 79:5367–81. doi: 10.1158/0008-5472.CAN-19-0454, PMID: 31439548

[48] Sun X Cai W Li H Gao C Ma X Guo Y. Endothelial-like cancer-associated fibroblasts facilitate pancreatic cancer metastasis via vasculogenic mimicry and paracrine signalling. Gut. (2025). doi: 10.1136/gutjnl-2024-333638, PMID: 40122596

[49] Krishnamurty AT Shyer JA Thai M Gandham V Buechler MB Yang YA. LRRC15(+) myofibroblasts dictate the stromal setpoint to suppress tumour immunity. Nature. (2022) 611:148–54. doi: 10.1038/s41586-022-05272-1, PMID: 36171287 PMC9630141

[50] Boyd LNC Andini KD Peters GJ Kazemier G Giovannetti E. Heterogeneity and plasticity of cancer-associated fibroblasts in the pancreatic tumor microenvironment. Semin Cancer Biol. (2022) 82:184–96. doi: 10.1016/j.semcancer.2021.03.006, PMID: 33737108

[51] Sheng N Shindo K Ohuchida K Shinkawa T Zhang B Feng H. TAK1 promotes an immunosuppressive tumor microenvironment through cancer-associated fibroblast phenotypic conversion in pancreatic ductal adenocarcinoma. Clin Cancer Res. (2024) 30:5138–53. doi: 10.1158/1078-0432.CCR-24-1004, PMID: 39264265 PMC11565170

[52] Schwörer S Cimino FV Ros M Tsanov KM Ng C Lowe SW. Hypoxia potentiates the inflammatory fibroblast phenotype promoted by pancreatic cancer cell-derived cytokines. Cancer Res. (2023) 83:1596–610. doi: 10.1158/0008-5472.Can-22-2316, PMID: 36912618 PMC10658995

[53] Feldmann K Maurer C Peschke K Teller S Schuck K Steiger K. Mesenchymal plasticity regulated by prrx1 drives aggressive pancreatic cancer biology. Gastroenterology. (2021) 160:346–61.e24. doi: 10.1053/j.gastro.2020.09.010, PMID: 33007300

[54] Steele NG Biffi G Kemp SB Zhang Y Drouillard D Syu L. Inhibition of hedgehog signaling alters fibroblast composition in pancreatic cancer. Clin Cancer Res. (2021) 27:2023–37. doi: 10.1158/1078-0432.CCR-20-3715, PMID: 33495315 PMC8026631

[55] Zaghdoudi S Decaup E Belhabib I Samain R Cassant-Sourdy S Rochotte J. FAK activity in cancer-associated fibroblasts is a prognostic marker and a druggable key metastatic player in pancreatic cancer. EMBO Mol Med. (2020) 12:e12010. doi: 10.15252/emmm.202012010, PMID: 33025708 PMC7645544

[56] Bockorny B Semenisty V Macarulla T Borazanci E Wolpin BM Stemmer SM. BL-8040, a CXCR4 antagonist, in combination with pembrolizumab and chemotherapy for pancreatic cancer: the COMBAT trial. Nat Med. (2020) 26:878–85. doi: 10.1038/s41591-020-0880-x, PMID: 32451495

[57] Tian C Clauser KR Öhlund D Rickelt S Huang Y Gupta M. Proteomic analyses of ECM during pancreatic ductal adenocarcinoma progression reveal different contributions by tumor and stromal cells. Proc Natl Acad Sci U.S.A. (2019) 116:19609–18. doi: 10.1073/pnas.1908626116, PMID: 31484774 PMC6765243

[58] Ashina S Masuda A Yamakawa K Hamada T Tsujimae M Tanaka T. A comprehensive analysis of tumor-stromal collagen in relation to pathological, molecular, and immune characteristics and patient survival in pancreatic ductal adenocarcinoma. J Gastroenterol. (2023) 58:1055–67. doi: 10.1007/s00535-023-02020-8, PMID: 37477731 PMC10522520

[59] Zheng JH Zhu YH Yang J Ji PX Zhao RK Duan ZH. A CLIC1 network coordinates matrix stiffness and the Warburg effect to promote tumor growth in pancreatic cancer. Cell Rep. (2024) 43:114633. doi: 10.1016/j.celrep.2024.114633, PMID: 39154343

[60] Liu Y Yao X Zhao Y Fang D Shi L Yang L. Mechanotransduction in response to ECM stiffening impairs cGAS immune signaling in tumor cells. Cell Rep. (2023) 42:113213. doi: 10.1016/j.celrep.2023.113213, PMID: 37804510

[61] Zhang T Chen J Yang H Sun X Ou Y Wang Q. Stromal softness confines pancreatic cancer growth through lysosomal-cathepsin mediated YAP1 degradation. Cell Mol Life Sci. (2024) 81:442. doi: 10.1007/s00018-024-05466-y, PMID: 39460766 PMC11512982

[62] Jiang H Torphy RJ Steiger K Hongo H Ritchie AJ Kriegsmann M. Pancreatic ductal adenocarcinoma progression is restrained by stromal matrix. J Clin Invest. (2020) 130:4704–9. doi: 10.1172/JCI136760, PMID: 32749238 PMC7456216

[63] Chen Y Kim J Yang S Wang H Wu CJ Sugimoto H. Type I collagen deletion in αSMA(+) myofibroblasts augments immune suppression and accelerates progression of pancreatic cancer. Cancer Cell. (2021) 39:548–65.e6. doi: 10.1016/j.ccell.2021.02.007, PMID: 33667385 PMC8423173

[64] Clementz AG Mutolo MJ Leir SH Morris KJ Kucybala K Harris H. Collagen XV inhibits epithelial to mesenchymal transition in pancreatic adenocarcinoma cells. PloS One. (2013) 8:e72250. doi: 10.1371/journal.pone.0072250, PMID: 23991074 PMC3750028

[65] Tian C Huang Y Clauser KR Rickelt S Lau AN Carr SA. Suppression of pancreatic ductal adenocarcinoma growth and metastasis by fibrillar collagens produced selectively by tumor cells. Nat Commun. (2021) 12:2328. doi: 10.1038/s41467-021-22490-9, PMID: 33879793 PMC8058088

[66] Tian C Öhlund D Rickelt S Lidström T Huang Y Hao L. Cancer cell-derived matrisome proteins promote metastasis in pancreatic ductal adenocarcinoma. Cancer Res. (2020) 80:1461–74. doi: 10.1158/0008-5472.CAN-19-2578, PMID: 32029550 PMC7127978

[67] Di Chiaro P Nacci L Arco F Brandini S Polletti S Palamidessi A. Mapping functional to morphological variation reveals the basis of regional extracellular matrix subversion and nerve invasion in pancreatic cancer. Cancer Cell. (2024) 42:662–81.e10. doi: 10.1016/j.ccell.2024.02.017, PMID: 38518775

[68] Kim EJ Sahai V Abel EV Griffith KA Greenson JK Takebe N. Pilot clinical trial of hedgehog pathway inhibitor GDC-0449 (vismodegib) in combination with gemcitabine in patients with metastatic pancreatic adenocarcinoma. Clin Cancer Res. (2014) 20:5937–45. doi: 10.1158/1078-0432.CCR-14-1269, PMID: 25278454 PMC4254161

[69] Folkman J. Tumor angiogenesis: therapeutic implications. N Engl J Med. (1971) 285:1182–6. doi: 10.1056/NEJM197111182852108, PMID: 4938153

[70] Jain RK. Normalization of tumor vasculature: an emerging concept in antiangiogenic therapy. Science. (2005) 307:58–62. doi: 10.1126/science.1104819, PMID: 15637262

[71] Rankin EB Giaccia AJ. The role of hypoxia-inducible factors in tumorigenesis. Cell Death Differ. (2008) 15:678–85. doi: 10.1038/cdd.2008.21, PMID: 18259193 PMC3050610

[72] Chen K Wang Q Liu X Wang F Yang Y Tian X. Hypoxic pancreatic cancer derived exosomal miR-30b-5p promotes tumor angiogenesis by inhibiting GJA1 expression. Int J Biol Sci. (2022) 18:1220–37. doi: 10.7150/ijbs.67675, PMID: 35173549 PMC8771853

[73] Shang D Xie C Hu J Tan J Yuan Y Liu Z. Pancreatic cancer cell-derived exosomal microRNA-27a promotes angiogenesis of human microvascular endothelial cells in pancreatic cancer via BTG2. J Cell Mol Med. (2020) 24:588–604. doi: 10.1111/jcmm.14766, PMID: 31724333 PMC6933412

[74] Adem B Bastos N Ruivo CF Sousa-Alves S Dias C Vieira PF. Exosomes define a local and systemic communication network in healthy pancreas and pancreatic ductal adenocarcinoma. Nat Commun. (2024) 15:1496. doi: 10.1038/s41467-024-45753-7, PMID: 38383468 PMC10881969

[75] Hendrix MJ Seftor EA Hess AR Seftor RE. Vasculogenic mimicry and tumour-cell plasticity: lessons from melanoma. Nat Rev Cancer. (2003) 3:411–21. doi: 10.1038/nrc1092, PMID: 12778131

[76] Kuczynski EA Vermeulen PB Pezzella F Kerbel RS Reynolds AR. Vessel co-option in cancer. Nat Rev Clin Oncol. (2019) 16:469–93. doi: 10.1038/s41571-019-0181-9, PMID: 30816337

[77] Benjakul N Prakobphol N Tangshewinsirikul C Dulyaphat W Svasti J Charngkaew K. Notch signaling regulates vasculogenic mimicry and promotes cell morphogenesis and the epithelial-to-mesenchymal transition in pancreatic ductal adenocarcinoma. PloS One. (2022) 17:e0279001. doi: 10.1371/journal.pone.0279001, PMID: 36548277 PMC9779037

[78] Zhuo M Yuan C Han T Hu H Cui J Jiao F. JQ1 effectively inhibits vasculogenic mimicry of pancreatic ductal adenocarcinoma cells via the ERK1/2-MMP-2/9 signaling pathway both *in vitro* and *in vivo* . Am J Transl Res. (2019) 11:1030–9., PMID: 30899402 PMC6413294

[79] Yang J Zhu DM Zhou XG Yin N Zhang Y Zhang ZX. HIF-2α promotes the formation of vasculogenic mimicry in pancreatic cancer by regulating the binding of Twist1 to the VE-cadherin promoter. Oncotarget. (2017) 8:47801–15. doi: 10.18632/oncotarget.17999, PMID: 28599281 PMC5564606

[80] Jiang Z Zhou J Li L Liao S He J Zhou S. Pericytes in the tumor microenvironment. Cancer Lett. (2023) 556:216074. doi: 10.1016/j.canlet.2023.216074, PMID: 36682706

[81] Kindler HL Niedzwiecki D Hollis D Sutherland S Schrag D Hurwitz H. Gemcitabine plus bevacizumab compared with gemcitabine plus placebo in patients with advanced pancreatic cancer: phase III trial of the Cancer and Leukemia Group B (CALGB 80303). J Clin Oncol. (2010) 28:3617–22. doi: 10.1200/JCO.2010.28.1386, PMID: 20606091 PMC2917317

[82] Stopczynski RE Normolle DP Hartman DJ Ying H Deberry JJ Bielefeldt K. Neuroplastic changes occur early in the development of pancreatic ductal adenocarcinoma. Cancer Res. (2014) 74:1718–27. doi: 10.1158/0008-5472.CAN-13-2050, PMID: 24448244 PMC4036226

[83] Bapat AA Hostetter G Von Hoff DD Han H. Perineural invasion and associated pain in pancreatic cancer. Nat Rev Cancer. (2011) 11:695–707. doi: 10.1038/nrc3131, PMID: 21941281

[84] Saloman JL Albers KM Rhim AD Davis BM. Can stopping nerves, stop cancer? Trends Neurosci. (2016) 39:880–9. doi: 10.1016/j.tins.2016.10.002, PMID: 27832915 PMC5148708

[85] Crippa S Pergolini I Javed AA Honselmann KC Weiss MJ Di SalvoF. Implications of perineural invasion on disease recurrence and survival after pancreatectomy for pancreatic head ductal adenocarcinoma. Ann Surg. (2022) 276:378–85. doi: 10.1097/SLA.0000000000004464, PMID: 33086324

[86] Saloman JL Singhi AD Hartman DJ Normolle DP Albers KM Davis BM. Systemic depletion of nerve growth factor inhibits disease progression in a genetically engineered model of pancreatic ductal adenocarcinoma. Pancreas. (2018) 47:856–63. doi: 10.1097/MPA.0000000000001090, PMID: 29975347 PMC6044729

[87] Zhu Z Friess H Dimola FF Zimmermann A Graber HU Korc M. Nerve growth factor expression correlates with perineural invasion and pain in human pancreatic cancer. J Clin Oncol. (1999) 17:2419–28. doi: 10.1200/JCO.1999.17.8.2419, PMID: 10561305

[88] Qin J Liu J Wei Z Li X Chen Z Li J. Targeted intervention in nerve-cancer crosstalk enhances pancreatic cancer chemotherapy. Nat Nanotechnol. (2024) 20(2):311–24. doi: 10.1038/s41565-024-01803-1, PMID: 39496914

[89] Thiel V Renders S Panten J Dross N Bauer K Azorin D. Characterization of single neurons reprogrammed by pancreatic cancer. Nature. (2025) 640:1042–51. doi: 10.1038/s41586-025-08735-3, PMID: 39961335 PMC12018453

[90] Hirth M Gandla J Höper C Gaida MM Agarwal N Simonetti M. CXCL10 and CCL21 promote migration of pancreatic cancer cells toward sensory neurons and neural remodeling in tumors in mice, associated with pain in patients. Gastroenterology. (2020) 159:665–81.e13. doi: 10.1053/j.gastro.2020.04.037, PMID: 32330476

[91] Globig AM Zhao S Roginsky J Maltez VI Guiza J Avina-Ochoa N. The β(1)-adrenergic receptor links sympathetic nerves to T cell exhaustion. Nature. (2023) 622:383–92. doi: 10.1038/s41586-023-06568-6, PMID: 37731001 PMC10871066

[92] Renz BW Tanaka T Sunagawa M Takahashi R Jiang Z Macchini M. Cholinergic signaling via muscarinic receptors directly and indirectly suppresses pancreatic tumorigenesis and cancer stemness. Cancer Discov. (2018) 8:1458–73. doi: 10.1158/2159-8290.CD-18-0046, PMID: 30185628 PMC6214763

[93] Yang MW Tao LY Jiang YS Yang JY Huo YM Liu DJ. Perineural invasion reprograms the immune microenvironment through cholinergic signaling in pancreatic ductal adenocarcinoma. Cancer Res. (2020) 80:1991–2003. doi: 10.1158/0008-5472.CAN-19-2689, PMID: 32098780

[94] Banh RS Biancur DE Yamamoto K Sohn ASW Walters B Kuljanin M. Neurons release serine to support mRNA translation in pancreatic cancer. Cell. (2020) 183:1202–18.e25. doi: 10.1016/j.cell.2020.10.016, PMID: 33142117 PMC8100789

[95] Li F He C Yao H Zhao Y Ye X Zhou S. Glutamate from nerve cells promotes perineural invasion in pancreatic cancer by regulating tumor glycolysis through HK2 mRNA-m6A modification. Pharmacol Res. (2023) 187:106555. doi: 10.1016/j.phrs.2022.106555, PMID: 36403721

[96] Deborde S Wong RJ. How Schwann cells facilitate cancer progression in nerves. Cell Mol Life Sci. (2017) 74:4405–20. doi: 10.1007/s00018-017-2578-x, PMID: 28631007 PMC5665723

[97] Deborde S Gusain L Powers A Marcadis A Yu Y Chen CH. Reprogrammed schwann cells organize into dynamic tracks that promote pancreatic cancer invasion. Cancer Discov. (2022) 12:2454–73. doi: 10.1158/2159-8290.CD-21-1690, PMID: 35881881 PMC9533012

[98] Tian Z Ou G Su M Li R Pan L Lin X. TIMP1 derived from pancreatic cancer cells stimulates Schwann cells and promotes the occurrence of perineural invasion. Cancer Lett. (2022) 546:215863. doi: 10.1016/j.canlet.2022.215863, PMID: 35961511

[99] Debnath J Gammoh N Ryan KM. Autophagy and autophagy-related pathways in cancer. Nat Rev Mol Cell Biol. (2023) 24:560–75. doi: 10.1038/s41580-023-00585-z, PMID: 36864290 PMC9980873

[100] Zhang W He R Yang W Zhang Y Yuan Q Wang J. Autophagic Schwann cells promote perineural invasion mediated by the NGF/ATG7 paracrine pathway in pancreatic cancer. J Exp Clin Cancer Res. (2022) 41:48. doi: 10.1186/s13046-021-02198-w, PMID: 35109895 PMC8809009

[101] Zheng S Hu C Lin Q. Extracellular vesicle-packaged PIAT from cancer-associated fibroblasts drives neural remodeling by mediating m5C modification in pancreatic cancer mouse models. Sci Transl Med. (2024) 16:eadi0178. doi: 10.1126/scitranslmed.adi0178, PMID: 39018369

[102] Li T Hu C Huang T Li T Li G Tian Q. Cancer-associated fibroblasts foster a high-lactate microenvironment to drive perineural invasion in pancreatic cancer. Cancer Res. (2025) 85(12): 2199–217. doi: 10.1158/0008-5472.c.7876842, PMID: 40138590 PMC12167935

[103] Hayashi A Hong J Iacobuzio-Donahue CA. The pancreatic cancer genome revisited. Nat Rev Gastroenterol Hepatol. (2021) 18:469–81. doi: 10.1038/s41575-021-00463-z, PMID: 34089011

[104] Liu Y Deguchi Y Wei D Liu F Moussalli MJ Deguchi E. Rapid acceleration of KRAS-mutant pancreatic carcinogenesis via remodeling of tumor immune microenvironment by PPARδ. Nat Commun. (2022) 13:2665. doi: 10.1038/s41467-022-30392-7, PMID: 35562376 PMC9106716

[105] Pylayeva-Gupta Y Lee KE Hajdu CH Miller G Bar-Sagi D. Oncogenic Kras-induced GM-CSF production promotes the development of pancreatic neoplasia. Cancer Cell. (2012) 21:836–47. doi: 10.1016/j.ccr.2012.04.024, PMID: 22698407 PMC3721510

[106] Caronni N La Terza F Vittoria FM Barbiera G Mezzanzanica L Cuzzola V. IL-1β+ macrophages fuel pathogenic inflammation in pancreatic cancer. Nature. (2023) 623:415–22. doi: 10.1038/s41586-023-06685-2, PMID: 37914939

[107] Gitto SB Beardsley JM Nakkina SP Oyer JL Cline KA Litherland SA. Identification of a novel IL-5 signaling pathway in chronic pancreatitis and crosstalk with pancreatic tumor cells. Cell Commun Signal. (2020) 18:95. doi: 10.1186/s12964-020-00594-x, PMID: 32552827 PMC7302008

[108] Mantovani A Allavena P Sica A Balkwill F. Cancer-related inflammation. Nature. (2008) 454:436–44. doi: 10.1038/nature07205, PMID: 18650914

[109] Chen Q Wang J Zhang Q Zhang J Lou Y Yang J. Tumour cell-derived debris and IgG synergistically promote metastasis of pancreatic cancer by inducing inflammation via tumour-associated macrophages. Br J Cancer. (2019) 121:786–95. doi: 10.1038/s41416-019-0595-2, PMID: 31588122 PMC6889176

[110] Cheng H Fan K Luo G Fan Z Yang C Huang Q. Kras(G12D) mutation contributes to regulatory T cell conversion through activation of the MEK/ERK pathway in pancreatic cancer. Cancer Lett. (2019) 446:103–11. doi: 10.1016/j.canlet.2019.01.013, PMID: 30664964

[111] Cheng H Luo G Jin K Fan Z Huang Q Gong Y. Kras mutation correlating with circulating regulatory T cells predicts the prognosis of advanced pancreatic cancer patients. Cancer Med. (2020) 9:2153–9. doi: 10.1002/cam4.2895, PMID: 32017404 PMC7064028

[112] Mahadevan KK Mcandrews KM Lebleu VS Yang S Lyu H Li B. KRAS(G12D) inhibition reprograms the microenvironment of early and advanced pancreatic cancer to promote FAS-mediated killing by CD8(+) T cells. Cancer Cell. (2023) 41:1606–20.e8. doi: 10.1016/j.ccell.2023.07.002, PMID: 37625401 PMC10785700

[113] Xie Y Zhou T Li X Zhao K Bai W Hou X. Targeting ESE3/EHF with nifurtimox inhibits CXCR2(+) neutrophil infiltration and overcomes pancreatic cancer resistance to chemotherapy and immunotherapy. Gastroenterology. (2024) 167:281–97. doi: 10.1053/j.gastro.2024.02.046, PMID: 38492894

[114] Zou S Zhang L Jiang C Li F Yang Y Deng X. Driver mutation subtypes involve with differentiated immunophenotypes influencing pancreatic cancer outcomes. Cancer Lett. (2024) 599:217134. doi: 10.1016/j.canlet.2024.217134, PMID: 39094824

[115] Ying H Kimmelman AC Lyssiotis CA Hua S Chu GC Fletcher-Sananikone E. Oncogenic Kras maintains pancreatic tumors through regulation of anabolic glucose metabolism. Cell. (2012) 149:656–70. doi: 10.1016/j.cell.2012.01.058, PMID: 22541435 PMC3472002

[116] Liberti MV Locasale JW. The warburg effect: how does it benefit cancer cells? Trends Biochem Sci. (2016) 41:211–8. doi: 10.1016/j.tibs.2015.12.001, PMID: 26778478 PMC4783224

[117] Li F Si W Xia L Yin D Wei T Tao M. Positive feedback regulation between glycolysis and histone lactylation drives oncogenesis in pancreatic ductal adenocarcinoma. Mol Cancer. (2024) 23:90. doi: 10.1186/s12943-024-02008-9, PMID: 38711083 PMC11071201

[118] Chen M Cen K Song Y Zhang X Liou YC Liu P. NUSAP1-LDHA-Glycolysis-Lactate feedforward loop promotes Warburg effect and metastasis in pancreatic ductal adenocarcinoma. Cancer Lett. (2023) 567:216285. doi: 10.1016/j.canlet.2023.216285, PMID: 37354982

[119] Encarnación-Rosado J Sohn ASW Biancur DE Lin EY Osorio-Vasquez V Rodrick T. Targeting pancreatic cancer metabolic dependencies through glutamine antagonism. Nat Cancer. (2024) 5:85–99. doi: 10.1038/s43018-023-00647-3, PMID: 37814010 PMC10824664

[120] Son J Lyssiotis CA Ying H Wang X Hua S Ligorio M. Glutamine supports pancreatic cancer growth through a KRAS-regulated metabolic pathway. Nature. (2013) 496:101–5. doi: 10.1038/nature12040, PMID: 23535601 PMC3656466

[121] Shen X Chen Y Tang Y Lu P Liu M Mao T. Targeting pancreatic cancer glutamine dependency confers vulnerability to GPX4-dependent ferroptosis. Cell Rep Med. (2025) 6:101928. doi: 10.1016/j.xcrm.2025.101928, PMID: 39879992 PMC11866519

[122] Bott AJ Shen J Tonelli C Zhan L Sivaram N Jiang YP. Glutamine anabolism plays a critical role in pancreatic cancer by coupling carbon and nitrogen metabolism. Cell Rep. (2019) 29:1287–98.e6. doi: 10.1016/j.celrep.2019.09.056, PMID: 31665640 PMC6886125

[123] Yin X Xu R Song J Ruze R Chen Y Wang C. Lipid metabolism in pancreatic cancer: emerging roles and potential targets. Cancer Commun (Lond). (2022) 42:1234–56. doi: 10.1002/cac2.12360, PMID: 36107801 PMC9759769

[124] García García A Ferrer Aporta M Vallejo Palma G Giráldez TrujilloA Castillo-González R Calzón LozanoD. Targeting ELOVL6 to disrupt c-MYC driven lipid metabolism in pancreatic cancer enhances chemosensitivity. Nat Commun. (2025) 16:1694. doi: 10.1038/s41467-025-56894-8, PMID: 39956817 PMC11830767

[125] Koutsioumpa M Hatziapostolou M Polytarchou C Tolosa EJ Almada LL Mahurkar-Joshi S. Lysine methyltransferase 2D regulates pancreatic carcinogenesis through metabolic reprogramming. Gut. (2019) 68:1271–86. doi: 10.1136/gutjnl-2017-315690, PMID: 30337373 PMC6697184

[126] Zhang B Ohuchida K Tsutsumi C Shimada Y Mochida Y Oyama K. Dynamic glycolytic reprogramming effects on dendritic cells in pancreatic ductal adenocarcinoma. J Exp Clin Cancer Res. (2024) 43:271. doi: 10.1186/s13046-024-03192-8, PMID: 39343933 PMC11441259

[127] Bharadwaj U Li M Zhang R Chen C Yao Q. Elevated interleukin-6 and G-CSF in human pancreatic cancer cell conditioned medium suppress dendritic cell differentiation and activation. Cancer Res. (2007) 67:5479–88. doi: 10.1158/0008-5472.CAN-06-3963, PMID: 17545630

[128] Liu Y Wang F Peng D Zhang D Liu L Wei J. Activation and antitumor immunity of CD8(+) T cells are supported by the glucose transporter GLUT10 and disrupted by lactic acid. Sci Transl Med. (2024) 16:eadk7399. doi: 10.1126/scitranslmed.adk7399, PMID: 39196962

[129] Lopez Krol A Nehring HP Krause FF Wempe A Raifer H Nist A. Lactate induces metabolic and epigenetic reprogramming of pro-inflammatory Th17 cells. EMBO Rep. (2022) 23:e54685. doi: 10.15252/embr.202254685, PMID: 36215678 PMC9724659

[130] Watson MJ Vignali PDA Mullett SJ Overacre-Delgoffe AE Peralta RM Grebinoski S. Metabolic support of tumour-infiltrating regulatory T cells by lactic acid. Nature. (2021) 591:645–51. doi: 10.1038/s41586-020-03045-2, PMID: 33589820 PMC7990682

[131] Kumagai S Koyama S Itahashi K Tanegashima T Lin YT Togashi Y. Lactic acid promotes PD-1 expression in regulatory T cells in highly glycolytic tumor microenvironments. Cancer Cell. (2022) 40:201–18.e9. doi: 10.1016/j.ccell.2022.01.001, PMID: 35090594

[132] Ding R Yu X Hu Z Dong Y Huang H Zhang Y. Lactate modulates RNA splicing to promote CTLA-4 expression in tumor-infiltrating regulatory T cells. Immunity. (2024) 57:528–40.e6. doi: 10.1016/j.immuni.2024.01.019, PMID: 38417442

[133] Dhar S Sarkar T Bose S Pati S Chakraborty D Roy D. FOXP3 transcriptionally activates fatty acid scavenger receptor CD36 in tumour-induced treg cells. Immunology. (2025) 174:296–309. doi: 10.1111/imm.13887, PMID: 39736083

[134] Xu S Chaudhary O Rodríguez-Morales P Sun X Chen D Zappasodi R. Uptake of oxidized lipids by the scavenger receptor CD36 promotes lipid peroxidation and dysfunction in CD8(+) T cells in tumors. Immunity. (2021) 54:1561–77.e7. doi: 10.1016/j.immuni.2021.05.003, PMID: 34102100 PMC9273026

[135] Colegio OR Chu NQ Szabo AL Chu T Rhebergen AM Jairam V. Functional polarization of tumour-associated macrophages by tumour-derived lactic acid. Nature. (2014) 513:559–63. doi: 10.1038/nature13490, PMID: 25043024 PMC4301845

[136] Sun K Zhang X Shi J Huang J Wang S Li X. Elevated protein lactylation promotes immunosuppressive microenvironment and therapeutic resistance in pancreatic ductal adenocarcinoma. J Clin Invest. (2025) 135(7). doi: 10.1172/JCI187024, PMID: 39883522 PMC11957693

[137] Wang L Tang W Yang S He P Wang J Gaedcke J. NO(•)/RUNX3/kynurenine metabolic signaling enhances disease aggressiveness in pancreatic cancer. Int J Cancer. (2020) 146:3160–9. doi: 10.1002/ijc.32733, PMID: 31609478 PMC8189162

[138] Hezaveh K Shinde RS Klötgen A Halaby MJ Lamorte S Ciudad MT. Tryptophan-derived microbial metabolites activate the aryl hydrocarbon receptor in tumor-associated macrophages to suppress anti-tumor immunity. Immunity. (2022) 55:324–40.e8. doi: 10.1016/j.immuni.2022.01.006, PMID: 35139353 PMC8888129

[139] Xiao J Wang S Chen L Ding X Dang Y Han M. 25-Hydroxycholesterol regulates lysosome AMP kinase activation and metabolic reprogramming to educate immunosuppressive macrophages. Immunity. (2024) 57:1087–104.e7. doi: 10.1016/j.immuni.2024.03.021, PMID: 38640930

[140] Tjomsland V Sandnes D Pomianowska E Cizmovic ST Aasrum M Brusevold IJ. The TGFβ-SMAD3 pathway inhibits IL-1α induced interactions between human pancreatic stellate cells and pancreatic carcinoma cells and restricts cancer cell migration. J Exp Clin Cancer Res. (2016) 35:122. doi: 10.1186/s13046-016-0400-5, PMID: 27473228 PMC4966589

[141] Tjomsland V Spångeus A Välilä J Sandström P Borch K Druid H. Interleukin 1α sustains the expression of inflammatory factors in human pancreatic cancer microenvironment by targeting cancer-associated fibroblasts. Neoplasia. (2011) 13:664–75. doi: 10.1593/neo.11332, PMID: 21847358 PMC3156657

[142] Sanford DE Belt BA Panni RZ Mayer A Deshpande AD Carpenter D. Inflammatory monocyte mobilization decreases patient survival in pancreatic cancer: a role for targeting the CCL2/CCR2 axis. Clin Cancer Res. (2013) 19:3404–15. doi: 10.1158/1078-0432.CCR-13-0525, PMID: 23653148 PMC3700620

[143] Bianchi A De Castro Silva I Deshpande NU Singh S Mehra S Garrido VT. Cell-autonomous cxcl1 sustains tolerogenic circuitries and stromal inflammation via neutrophil-derived TNF in pancreatic cancer. Cancer Discov. (2023) 13:1428–53. doi: 10.1158/2159-8290.CD-22-1046, PMID: 36946782 PMC10259764

[144] Nemunaitis J. Vaccines in cancer: GVAX, a GM-CSF gene vaccine. Expert Rev Vaccines. (2005) 4:259–74. doi: 10.1586/14760584.4.3.259, PMID: 16026242

[145] Boelaars K Rodriguez E Huinen ZR Liu C Wang D Springer BO. Pancreatic cancer-associated fibroblasts modulate macrophage differentiation via sialic acid-Siglec interactions. Commun Biol. (2024) 7:430. doi: 10.1038/s42003-024-06087-8, PMID: 38594506 PMC11003967

[146] Lee BY Hogg EKJ Below CR Kononov A Blanco-Gomez A Heider F. Heterocellular OSM-OSMR signalling reprograms fibroblasts to promote pancreatic cancer growth and metastasis. Nat Commun. (2021) 12:7336. doi: 10.1038/s41467-021-27607-8, PMID: 34921158 PMC8683436

[147] Albrengues J Shields MA Ng D Park CG Ambrico A Poindexter ME. Neutrophil extracellular traps produced during inflammation awaken dormant cancer cells in mice. Science. (2018) 361(6409). doi: 10.1126/science.aao4227, PMID: 30262472 PMC6777850

[148] Munir H Jones JO Janowitz T Hoffmann M Euler M Martins CP. Stromal-driven and Amyloid β-dependent induction of neutrophil extracellular traps modulates tumor growth. Nat Commun. (2021) 12:683. doi: 10.1038/s41467-021-20982-2, PMID: 33514748 PMC7846803

[149] Chen M Zhang Y Zhou P Liu X Zhao H Zhou X. Substrate stiffness modulates bone marrow-derived macrophage polarization through NF-κB signaling pathway. Bioact Mater. (2020) 5:880–90. doi: 10.1016/j.bioactmat.2020.05.004, PMID: 32637751 PMC7332470

[150] Feig C Jones JO Kraman M Wells RJ Deonarine A Chan DS. Targeting CXCL12 from FAP-expressing carcinoma-associated fibroblasts synergizes with anti-PD-L1 immunotherapy in pancreatic cancer. Proc Natl Acad Sci U.S.A. (2013) 110:20212–7. doi: 10.1073/pnas.1320318110, PMID: 24277834 PMC3864274

[151] Xiao Z Todd L Huang L Noguera-Ortega E Lu Z Huang L. Desmoplastic stroma restricts T cell extravasation and mediates immune exclusion and immunosuppression in solid tumors. Nat Commun. (2023) 14:5110. doi: 10.1038/s41467-023-40850-5, PMID: 37607999 PMC10444764

[152] Zhang J Li J Hou Y Lin Y Zhao H Shi Y. Osr2 functions as a biomechanical checkpoint to aggravate CD8(+) T cell exhaustion in tumor. Cell. (2024) 187(13):3409–26. e24. doi: 10.1016/j.cell.2024.04.023, PMID: 38744281

[153] Liu Y Sinjab A Min J Han G Paradiso F Zhang Y. Conserved spatial subtypes and cellular neighborhoods of cancer-associated fibroblasts revealed by single-cell spatial multi-omics. Cancer Cell. (2025) 43:905–24.e6. doi: 10.1016/j.ccell.2025.03.004, PMID: 40154487 PMC12074878

[154] Assouline B Kahn R Hodali L Condiotti R Engel Y Elyada E. Senescent cancer-associated fibroblasts in pancreatic adenocarcinoma restrict CD8(+) T cell activation and limit responsiveness to immunotherapy in mice. Nat Commun. (2024) 15:6162. doi: 10.1038/s41467-024-50441-7, PMID: 39039076 PMC11263607

[155] Wang K Ni B Xie Y Li Z Yuan L Meng C. Nociceptor neurons promote PDAC progression and cancer pain by interaction with cancer-associated fibroblasts and suppression of natural killer cells. Cell Res. (2025) 35:362–80. doi: 10.1038/s41422-025-01098-4, PMID: 40122998 PMC12012126

[156] Bausch D Fritz S Bolm L Wellner UF Fernandez-Del-Castillo C Warshaw AL. Hedgehog signaling promotes angiogenesis directly and indirectly in pancreatic cancer. Angiogenesis. (2020) 23:479–92. doi: 10.1007/s10456-020-09725-x, PMID: 32444947

[157] Yang Y Guo Z Chen W Wang X Cao M Han X. M2 macrophage-derived exosomes promote angiogenesis and growth of pancreatic ductal adenocarcinoma by targeting E2F2. Mol Ther. (2021) 29:1226–38. doi: 10.1016/j.ymthe.2020.11.024, PMID: 33221435 PMC7934635

[158] Liu Y Cao X. Characteristics and significance of the pre-metastatic niche. Cancer Cell. (2016) 30:668–81. doi: 10.1016/j.ccell.2016.09.011, PMID: 27846389

[159] Yachida S Iacobuzio-Donahue CA. The pathology and genetics of metastatic pancreatic cancer. Arch Pathol Lab Med. (2009) 133:413–22. doi: 10.5858/133.3.413, PMID: 19260747

[160] Lee JW Stone ML Porrett PM Thomas SK Komar CA Li JH. Hepatocytes direct the formation of a pro-metastatic niche in the liver. Nature. (2019) 567:249–52. doi: 10.1038/s41586-019-1004-y, PMID: 30842658 PMC6430113

[161] Ho WJ Erbe R Danilova L Phyo Z Bigelow E Stein-O'brien G. Multi-omic profiling of lung and liver tumor microenvironments of metastatic pancreatic cancer reveals site-specific immune regulatory pathways. Genome Biol. (2021) 22:154. doi: 10.1186/s13059-021-02363-6, PMID: 33985562 PMC8118107

[162] Luckett T Abudula M Ireland L Glenn M Bellomo G Stafferton R. Mesothelin secretion by pancreatic cancer cells co-opts macrophages and promotes metastasis. Cancer Res. (2024) 84:527–44. doi: 10.1158/0008-5472.CAN-23-1542, PMID: 38356443

[163] Thomas SK Wattenberg MM Choi-Bose S Uhlik M Harrison B Coho H. Kupffer cells prevent pancreatic ductal adenocarcinoma metastasis to the liver in mice. Nat Commun. (2023) 14:6330. doi: 10.1038/s41467-023-41771-z, PMID: 37816712 PMC10564762

[164] O’reilly EM Oh DY Dhani N Renouf DJ Lee MA Sun W. Durvalumab with or without tremelimumab for patients with metastatic pancreatic ductal adenocarcinoma: A phase 2 randomized clinical trial. JAMA Oncol. (2019) 5:1431–8. doi: 10.1001/jamaoncol.2019.1588, PMID: 31318392 PMC6647002

[165] Kaur A Riaz MS Murugaiah V Varghese PM Singh SK Kishore U. A Recombinant Fragment of Human Surfactant Protein D induces Apoptosis in Pancreatic Cancer Cell Lines via Fas-Mediated Pathway. Front Immunol. (2018) 9:1126. doi: 10.3389/fimmu.2018.01126, PMID: 29915574 PMC5994421

[166] Kaur A Riaz MS Singh SK Kishore U. Human surfactant protein D suppresses epithelial-to-mesenchymal transition in pancreatic cancer cells by downregulating TGF-β. Front Immunol. (2018) 9:1844. doi: 10.3389/fimmu.2018.01844, PMID: 30158928 PMC6104167

[167] Wei Q Ye Z Zhong X Li L Wang C Myers RE. Multiregion whole-exome sequencing of matched primary and metastatic tumors revealed genomic heterogeneity and suggested polyclonal seeding in colorectal cancer metastasis. Ann Oncol. (2017) 28:2135–41. doi: 10.1093/annonc/mdx278, PMID: 28911083 PMC5834069

[168] Ghebrehiwet B Zaniewski M Fernandez A Digiovanni M Reyes TN Ji P. The C1q and gC1qR axis as a novel checkpoint inhibitor in cancer. Front Immunol. (2024) 15:1351656. doi: 10.3389/fimmu.2024.1351656, PMID: 38711524 PMC11070495

[169] Aykut B Pushalkar S Chen R Li Q Abengozar R Kim JI. The fungal mycobiome promotes pancreatic oncogenesis via activation of MBL. Nature. (2019) 574:264–7. doi: 10.1038/s41586-019-1608-2, PMID: 31578522 PMC6858566

[170] Van Niel G D’angelo G Raposo G. Shedding light on the cell biology of extracellular vesicles. Nat Rev Mol Cell Biol. (2018) 19:213–28. doi: 10.1038/nrm.2017.125, PMID: 29339798

[171] Li Q He G Yu Y Li Q Abengozar R Kim JI. Exosome crosstalk between cancer stem cells and tumor microenvironment: cancer progression and therapeutic strategies. Stem Cell Res Ther. (2024) 15:449. doi: 10.1186/s13287-024-04061-z, PMID: 39578849 PMC11583673

[172] Kugeratski FG Kalluri R. Exosomes as mediators of immune regulation and immunotherapy in cancer. FEBS J. (2021) 288:10–35. doi: 10.1111/febs.15558, PMID: 32910536 PMC9116040

[173] Zhou M Chen J Zhou L Chen W Ding G Cao L. Pancreatic cancer derived exosomes regulate the expression of TLR4 in dendritic cells via miR-203. Cell Immunol. (2014) 292:65–9. doi: 10.1016/j.cellimm.2014.09.004, PMID: 25290620

[174] Dai E Han L Liu J Xie Y Kroemer G Klionsky DJ. Autophagy-dependent ferroptosis drives tumor-associated macrophage polarization via release and uptake of oncogenic KRAS protein. Autophagy. (2020) 16:2069–83. doi: 10.1080/15548627.2020.1714209, PMID: 31920150 PMC7595620

[175] Wang X Luo G Zhang K Cao J Huang C Jiang T. Hypoxic Tumor-Derived Exosomal miR-301a Mediates M2 Macrophage Polarization via PTEN/PI3Kγ to Promote Pancreatic Cancer Metastasis. Cancer Res. (2018) 78:4586–98. doi: 10.1158/0008-5472.CAN-17-3841, PMID: 29880482

[176] Mu W Xu Y Gu P Wang W Li J Ge Y. Exosomal CD44 cooperates with integrin α6β4 to support organotropic metastasis via regulating tumor cell motility and target host cell activation. Engineering. (2021) 7:1413–23. doi: 10.1016/j.eng.2020.08.013

[177] Xie Z Gao Y Ho C Li L Jin C Wang X. Exosome-delivered CD44v6/C1QBP complex drives pancreatic cancer liver metastasis by promoting fibrotic liver microenvironment. Gut. (2022) 71:568–79. doi: 10.1136/gutjnl-2020-323014, PMID: 33827783

[178] Chen W Peng W Wang R Bai S Cao M Xiong S. Exosome-derived tRNA fragments tRF-GluCTC-0005 promotes pancreatic cancer liver metastasis by activating hepatic stellate cells. Cell Death Dis. (2024) 15:102. doi: 10.1038/s41419-024-06482-3, PMID: 38291031 PMC10827722

[179] Cao W Dai S Ruan W Long T Zeng Z Lei S. Pancreatic stellate cell-derived exosomal tRF-19-PNR8YPJZ promotes proliferation and mobility of pancreatic cancer through AXIN2. J Cell Mol Med. (2023) 27:2533–46. doi: 10.1111/jcmm.17852, PMID: 37488774 PMC10468654

[180] Li M Guo H Wang Q Chen K Marko K Tian X. Pancreatic stellate cells derived exosomal miR-5703 promotes pancreatic cancer by downregulating CMTM4 and activating PI3K/Akt pathway. Cancer Lett. (2020) 490:20–30. doi: 10.1016/j.canlet.2020.06.009, PMID: 32585413

[181] Qin C Zhao B Wang Y Li Z Li T Zhao Y. Extracellular vesicles miR-31-5p promotes pancreatic cancer chemoresistance via regulating LATS2-Hippo pathway and promoting SPARC secretion from pancreatic stellate cells. J Extracell Vesicles. (2024) 13:e12488. doi: 10.1002/jev2.12488, PMID: 39104296 PMC11300957

[182] Chi Y Xin H Liu Z. Exosomal lncRNA UCA1 Derived From Pancreatic Stellate Cells Promotes Gemcitabine Resistance in Pancreatic Cancer via the SOCS3/EZH2 Axis. Front Oncol. (2021) 11:671082. doi: 10.3389/fonc.2021.671082, PMID: 34868904 PMC8640181

[183] Qi R Bai Y Li K Liu N Xu Y Dal E. Cancer-associated fibroblasts suppress ferroptosis and induce gemcitabine resistance in pancreatic cancer cells by secreting exosome-derived ACSL4-targeting miRNAs. Drug Resist Update. (2023) 68:100960. doi: 10.1016/j.drup.2023.100960, PMID: 37003125

[184] Richards KE Xiao W Hill R On Behalf of the Usc Pancreas Research T. Cancer-Associated Fibroblasts Confer Gemcitabine Resistance to Pancreatic Cancer Cells through PTEN-Targeting miRNAs in Exosomes. Cancers (Basel). (2022) 14(11). doi: 10.3390/cancers14112812, PMID: 35681792 PMC9179363

[185] Yao H Huang C Zou J Liang W Zhao Y Yang K. Extracellular vesicle-packaged lncRNA from cancer-associated fibroblasts promotes immune evasion by downregulating HLA-A in pancreatic cancer. J Extracell Vesicles. (2024) 13:e12484. doi: 10.1002/jev2.12484, PMID: 39041344 PMC11263977

[186] Liu F Chen J Li K Li H Zhu Y Zhai Y. Ubiquitination and deubiquitination in cancer: from mechanisms to novel therapeutic approaches. Mol Cancer. (2024) 23:148. doi: 10.1186/s12943-024-02046-3, PMID: 39048965 PMC11270804

[187] Zhang K Li YJ Peng LJ Gao HF Liu LM Chen H. M2 macrophage-derived exosomal miR-193b-3p promotes progression and glutamine uptake of pancreatic cancer by targeting TRIM62. Biol Direct. (2023) 18:1. doi: 10.1186/s13062-023-00356-y, PMID: 36631876 PMC9832623

[188] Yin Z Ma T Huang B Lin L Zhou Y Yan J. Macrophage-derived exosomal microRNA-501-3p promotes progression of pancreatic ductal adenocarcinoma through the TGFBR3-mediated TGF-β signaling pathway. J Exp Clin Cancer Res. (2019) 38:310. doi: 10.1186/s13046-019-1313-x, PMID: 31307515 PMC6631643

[189] Jiang H Hegde S Knolhoff BL Zhu Y Herndon JM Meyer MA. Targeting focal adhesion kinase renders pancreatic cancers responsive to checkpoint immunotherapy. Nat Med. (2016) 22:851–60. doi: 10.1038/nm.4123, PMID: 27376576 PMC4935930

[190] Van Cutsem E Tempero MA Sigal D Oh DY Fazio N Macarulla T. Randomized phase III trial of pegvorhyaluronidase alfa with nab-paclitaxel plus gemcitabine for patients with hyaluronan-high metastatic pancreatic adenocarcinoma. J Clin Oncol. (2020) 38:3185–94. doi: 10.1200/JCO.20.00590, PMID: 32706635 PMC7499614

[191] Ramanathan RK Mcdonough SL Philip PA Hingorani SR Lacy J Kortmansky JS. Phase IB/II randomized study of FOLFIRINOX plus pegylated recombinant human hyaluronidase versus FOLFIRINOX alone in patients with metastatic pancreatic adenocarcinoma: SWOG S1313. J Clin Oncol. (2019) 37:1062–9. doi: 10.1200/JCO.18.01295, PMID: 30817250 PMC6494359

[192] Ko AH Kim KP Siveke JT Lopez CD Lacy J O'reilly EM. Atezolizumab plus PEGPH20 versus chemotherapy in advanced pancreatic ductal adenocarcinoma and gastric cancer: MORPHEUS phase ib/II umbrella randomized study platform. Oncologist. (2023) 28:553–e472. doi: 10.1093/oncolo/oyad022, PMID: 36940261 PMC10243783

[193] Wehrli M Guinn S Birocchi F Kuo A Sun Y Larson RC. Mesothelin CAR T cells secreting anti-FAP/anti-CD3 molecules efficiently target pancreatic adenocarcinoma and its stroma. Clin Cancer Res. (2024) 30:1859–77. doi: 10.1158/1078-0432.CCR-23-3841, PMID: 38393682 PMC11062832

[194] Zhang X Lao M Yang H Sun K Dong Y He L. Targeting cancer-associated fibroblast autophagy renders pancreatic cancer eradicable with immunochemotherapy by inhibiting adaptive immune resistance. Autophagy. (2024) 20:1314–34. doi: 10.1080/15548627.2023.2300913, PMID: 38174993 PMC11210910

[195] Lesavage BL Zhang D Huerta-López C Gilchrist AE Krajina BA Karlsson K. Engineered matrices reveal stiffness-mediated chemoresistance in patient-derived pancreatic cancer organoids. Nat Mater. (2024) 23:1138–49. doi: 10.1038/s41563-024-01908-x, PMID: 38965405 PMC13098013

[196] Gunderson AJ Kaneda MM Tsujikawa T Nguyen AV Affara NI Ruffell B. Bruton tyrosine kinase-dependent immune cell cross-talk drives pancreas cancer. Cancer Discov. (2016) 6:270–85. doi: 10.1158/2159-8290.CD-15-0827, PMID: 26715645 PMC4783268

[197] Das S Bar-Sagi D. BTK signaling drives CD1d(hi)CD5(+) regulatory B-cell differentiation to promote pancreatic carcinogenesis. Oncogene. (2019) 38:3316–24. doi: 10.1038/s41388-018-0668-3, PMID: 30635655 PMC6486434

[198] Barry ST Gabrilovich DI Sansom OJ Campbell AD Morton JP. Therapeutic targeting of tumour myeloid cells. Nat Rev Cancer. (2023) 23:216–37. doi: 10.1038/s41568-022-00546-2, PMID: 36747021

[199] Kebenko M Goebeler ME Wolf M Hasenburg A Seggewiss-Bernhardt R Ritter B. A multicenter phase 1 study of solitomab (MT110, AMG 110), a bispecific EpCAM/CD3 T-cell engager (BiTE^®^) antibody construct, in patients with refractory solid tumors. Oncoimmunology. (2018) 7:e1450710. doi: 10.1080/2162402X.2018.1450710, PMID: 30221040 PMC6136859

[200] Voltà-Durán E Alba-Castellón L Serna N Casanova I López-Laguna H Gallardo A. High-precision targeting and destruction of cancer-associated PDGFR-β(+) stromal fibroblasts through self-assembling, protein-only nanoparticles. Acta Biomater. (2023) 170:543–55. doi: 10.1016/j.actbio.2023.09.001, PMID: 37683965

[201] Zhao J Wang H Hsiao CH Chow DS Koay EJ Kang Y. Simultaneous inhibition of hedgehog signaling and tumor proliferation remodels stroma and enhances pancreatic cancer therapy. Biomaterials. (2018) 159:215–28. doi: 10.1016/j.biomaterials.2018.01.014, PMID: 29331808 PMC6203960

[202] Yang P Xu Y Zhi X Li R Wang B Liu R. Photodynamically tumor vessel destruction amplified tumor targeting of nanoparticles for efficient chemotherapy. ACS Nano. (2024) 18:12933–44. doi: 10.1021/acsnano.4c00833, PMID: 38712906

[203] Park JJH Hsu G Siden EG Thorlund K Mills EJ. An overview of precision oncology basket and umbrella trials for clinicians. CA Cancer J Clin. (2020) 70:125–37. doi: 10.3322/caac.21600, PMID: 32031692 PMC7187272

[204] Canel M Sławińska AD Lonergan DW Kallor AA Upstill-Goddard R Davidson C. FAK suppresses antigen processing and presentation to promote immune evasion in pancreatic cancer. Gut. (2023) 73:131–55. doi: 10.1136/gutjnl-2022-327927, PMID: 36977556 PMC10715489

[205] Bendell J Sharma S Patel MR Windsor KS Wainberg ZA Gordon M. Safety and efficacy of andecaliximab (GS-5745) plus gemcitabine and nab-paclitaxel in patients with advanced pancreatic adenocarcinoma: results from a phase I study. Oncologist. (2020) 25:954–62. doi: 10.1634/theoncologist.2020-0474, PMID: 32812320 PMC7648353

[206] Tekin C Aberson HL Waasdorp C Hooijer GKJ De BoerOJ Dijk F. Macrophage-secreted MMP9 induces mesenchymal transition in pancreatic cancer cells via PAR1 activation. Cell Oncol (Dordr). (2020) 43:1161–74. doi: 10.1007/s13402-020-00549-x, PMID: 32809114 PMC7717035

[207] Raymant M Astuti Y Alvaro-Espinosa L Green D Quaranta V Bellomo G. Macrophage-fibroblast JAK/STAT dependent crosstalk promotes liver metastatic outgrowth in pancreatic cancer. Nat Commun. (2024) 15:3593. doi: 10.1038/s41467-024-47949-3, PMID: 38678021 PMC11055860

[208] Biffi G Oni TE Spielman B Hao Y Elyada E Park Y. IL1-induced JAK/STAT signaling is antagonized by TGFβ to shape CAF heterogeneity in pancreatic ductal adenocarcinoma. Cancer Discov. (2019) 9:282–301. doi: 10.1158/2159-8290.CD-18-0710, PMID: 30366930 PMC6368881

[209] Pérez-Gutiérrez L Ferrara N. Biology and therapeutic targeting of vascular endothelial growth factor A. Nat Rev Mol Cell Biol. (2023) 24:816–34. doi: 10.1038/s41580-023-00631-w, PMID: 37491579

[210] Shaib WL Manali R Liu Y El-Rayes B Loehrer P O'neil B. Phase II randomised, double-blind study of mFOLFIRINOX plus ramucirumab versus mFOLFIRINOX plus placebo in advanced pancreatic cancer patients (HCRN GI14-198). Eur J Cancer. (2023) 189:112847. doi: 10.1016/j.ejca.2023.02.030, PMID: 37268519

[211] Zhang X Liu Y Zhang Z Tan J Zhang J Ou H. Multi-omics analysis of anlotinib in pancreatic cancer and development of an anlotinib-related prognostic signature[J. Front Cell Dev Biol. (2021) 9:649265. doi: 10.3389/fcell.2021.649265, PMID: 33748143 PMC7969999

[212] Qiu X Lu C Sha H Zhu Y Kong W Tong F. Efficacy and safety of second-line therapy by S-1 combined with sintilimab and anlotinib in pancreatic cancer patients with liver metastasis: a single-arm, phase II clinical trial. Front Immunol. (2024) 15:1210859. doi: 10.3389/fimmu.2024.1210859, PMID: 38361920 PMC10867188

[213] Steele CW Karim SA Leach JDG Bailey P Upstill-Goddard R Rishi L. CXCR2 inhibition profoundly suppresses metastases and augments immunotherapy in pancreatic ductal adenocarcinoma. Cancer Cell. (2016) 29:832–45. doi: 10.1016/j.ccell.2016.04.014, PMID: 27265504 PMC4912354

[214] Heumann T Judkins C Li K Lim SJ Hoare J Parkinson R. A platform trial of neoadjuvant and adjuvant antitumor vaccination alone or in combination with PD-1 antagonist and CD137 agonist antibodies in patients with resectable pancreatic adenocarcinoma. Nat Commun. (2023) 14:3650. doi: 10.1038/s41467-023-39196-9, PMID: 37339979 PMC10281953

[215] Muth ST Saung MT Blair AB Henderson MG Thomas DL Zheng L. CD137 agonist-based combination immunotherapy enhances activated, effector memory T cells and prolongs survival in pancreatic adenocarcinoma. Cancer Lett. (2021) 499:99–108. doi: 10.1016/j.canlet.2020.11.041, PMID: 33271264 PMC7779747

[216] Chen Z Zhang S Han N Jiang J Xu Y Ma D. A neoantigen-based peptide vaccine for patients with advanced pancreatic cancer refractory to standard treatment. Front Immunol. (2021) 12:691605. doi: 10.3389/fimmu.2021.691605, PMID: 34484187 PMC8414362

[217] Jo JH Kim YT Choi HS Kim HG Lee HS Choi YW. Efficacy of GV1001 with gemcitabine/capecitabine in previously untreated patients with advanced pancreatic ductal adenocarcinoma having high serum eotaxin levels (KG4/2015): an open-label, randomised, Phase 3 trial. Br J Cancer. (2024) 130:43–52. doi: 10.1038/s41416-023-02474-w, PMID: 37903909 PMC10781743

[218] Beatty GL Chiorean EG Fishman MP Saboury B Teitelbaum UR Sun W. CD40 agonists alter tumor stroma and show efficacy against pancreatic carcinoma in mice and humans. Science. (2011) 331:1612–6. doi: 10.1126/science.1198443, PMID: 21436454 PMC3406187

[219] Morrison AH Diamond MS Hay CA Byrne KT Vonderheide RH. Sufficiency of CD40 activation and immune checkpoint blockade for T cell priming and tumor immunity. Proc Natl Acad Sci U.S.A. (2020) 117:8022–31. doi: 10.1073/pnas.1918971117, PMID: 32213589 PMC7149500

